# FGFR2b signalling restricts lineage-flexible alveolar progenitors during mouse lung development and converges in mature alveolar type 2 cells

**DOI:** 10.1007/s00018-022-04626-2

**Published:** 2022-11-29

**Authors:** Matthew R. Jones, Arun Lingampally, Negah Ahmadvand, Lei Chong, Jin Wu, Jochen Wilhem, Ana Ivonne Vazquez-Armendariz, Meshal Ansari, Susanne Herold, David M. Ornitz, Herbert B. Schiller, Cho-Ming Chao, Jin-San Zhang, Gianni Carraro, Saverio Bellusci

**Affiliations:** 1 Cardio-Pulmonary Institute (CPI), Universities of Giessen and Marburg Lung Center (UGMLC), German Center for Lung Research (DZL), Justus-Liebig University Giessen, Giessen, Germany; 2 China National Key Clinical Specialty of Pediatric Respiratory Medicine, Institute of Pediatrics, The Second Affiliated Hospital and Yuying Children′s Hospital of Wenzhou Medical University, Wenzhou, 325027 Zhejiang China; 3 Department of Pulmonary and Critical Care Medicine, The First Affiliated Hospital of Wenzhou Medical University, Wenzhou, Zhejiang China; 4 Institute of Lung Health (ILH), Giessen, Germany; 5 Department of Medicine V, Internal Medicine, Infectious Diseases and Infection Control, Universities of Giessen and Marburg Lung Center (UGMLC), German Center for Lung Research (DZL), Justus-Liebig University Giessen, Giessen, Germany; 6 Institute of Lung Biology and Disease and Comprehensive Pneumology Center, German Center for Lung Research (DZL), Helmholtz Zentrum Munchen, Munich, Germany; 7 Department of Developmental Biology, Washington University School of Medicine, 660 S. Euclid Avenue, St. Louis, MO 63110 USA; 8 Center for Child and Adolescent Medicine, Centre for Clinical and Translational Research (CCTR), Helios University Hospital Wuppertal, Witten/Herdecke University, 42283 Wuppertal, Germany; 9 The Quzhou Affiliated Hospital of Wenzhou Medical University, Quzhou People′s Hospital, 324000 Quzhou, Zhejiang China; 10 Department of Medicine, Cedars-Sinai Medical Center, Lung and Regenerative Medicine Institutes, Los Angeles, CA USA; 11 Laboratory of Extracellular Lung Matrix Remodelling, Department of Internal Medicine, Cardio-Pulmonary Institute and Institute for Lung Health, Universities of Giessen and Marburg Lung Center (UGMLC), German Center for Lung Research (DZL), Justus-Liebig University Giessen, 35392 Giessen, Germany

**Keywords:** Fgfr2b, Alveolar type 1, Alveolar type 2, Lung development, Alveolar lineage

## Abstract

The specification, characterization, and fate of alveolar type 1 and type 2 (AT1 and AT2) progenitors during embryonic lung development are poorly defined. Current models of distal epithelial lineage formation fail to capture the heterogeneity and dynamic contribution of progenitor pools present during early development. Furthermore, few studies explore the pathways involved in alveolar progenitor specification and fate. In this paper, we build upon our previously published work on the regulation of airway epithelial progenitors by fibroblast growth factor receptor 2b (FGFR2b) signalling during early (E12.5) and mid (E14.5) pseudoglandular stage lung development. Our results suggest that a significant proportion of AT2 and AT1 progenitors are lineage-flexible during late pseudoglandular stage development, and that lineage commitment is regulated in part by FGFR2b signalling. We have characterized a set of direct FGFR2b targets at E16.5 which are likely involved in alveolar lineage formation. These signature genes converge on a subpopulation of AT2 cells later in development and are downregulated in AT2 cells transitioning to the AT1 lineage during repair after injury in adults. Our findings highlight the extensive heterogeneity of pneumocytes by elucidating the role of FGFR2b signalling in these cells during early airway epithelial lineage formation, as well as during repair after injury.

**Supplementary Information:**

The online version contains supplementary material available at 10.1007/s00018-022-04626-2.

## Introduction

Around embryonic day (E) 9.5 in the mouse, a specialized region of *Nkx2-1* expressing endoderm of the anterior foregut evaginates, forming the anlage of the trachea and lung. During the ensuing embryonic and pseudoglandular stages of development (E9.5–E16.5), the growing multipotent epithelium undergoes branching morphogenesis, concomitant with the earliest differentiation and cell-fate specification of the various epithelial cell types. The developing epithelium quickly resembles a highly branched tree of proximal conducting airways, demarcated by SOX2 expression, and distal buds positive for SOX9 and ID2 [1]. By the end of pseudoglandular stage development, and leading into the canalicular (E16.5–E17.5) and saccular stages [E17.5–postnatal (PN) day 4], distal progenitors expressing markers for alveolar epithelial type 1 (AT1) and type 2 (AT2) cell lineages populate the nascent alveoli. Essential for proper lung function, AT1 cell bodies form thin elongated extensions lining the apical sides of alveoli, providing a gas exchange interface between the alveolar lumen and the capillary plexus located on the basal epithelial surface; AT2 cells are cuboidal, are distributed around the perimeters of alveoli, and produce and secrete surfactant, which reduces epithelial surface tension thus preventing alveolar collapse. AT2 cells are also considered as stem cells, capable of self-renewal or differentiation to AT1s upon repair after injury [2].

The cellular mechanisms by which *Nkx2-1* positive multipotent progenitors progress towards a distal epithelial lineage specification and eventual commitment to an AT1 or AT2 cell fate are poorly defined. A complex interplay of signalling pathways and biomechanical and physical factors regulates the spatial and temporal emergence of progenitor cells during development [3–5]. However, it is still unclear when the alveolar lineages proper emerge, and what are the characteristics of the progenitor cell(s). This uncertainty is captured by two different, yet not necessarily exclusive, models of pneumocyte lineage specification: the bipotent progenitor model, and the early lineage specification model.

In the first, earlier model, it was proposed that oligopotent distal epithelial progenitors give rise to a progenitor population called “bipotent progenitor cells (BPs)”. It was argued that these BPs, which are detected at E16.5 at the end of the branching program, can self-renew or differentiate into either the AT1 (expressing the canonical marker *Pdpn*) or AT2 lineage (expressing the canonical markers *Sftpc* and *Sftpb*). Based on single-cell transcriptomic studies of the epithelium at different stages during lung development, a gene signature characteristic of each differentiated cell type was identified. BPs exhibit both AT1 and AT2 signatures, and, during differentiation, progressively lose one or the other signature to become a mature alveolar cell [6].

An important limitation of the model placing BPs as the hub for both AT1 and AT2 formation was that lineage tracing approaches for these cells were missing. In the second and more recent model, which did combine single-cell transcriptomics along with lineage tracing, it was proposed that most mature AT1 and AT2 cells arise from unipotent progenitors, largely bypassing the BP cell type [7]. Furthermore, it was suggested that committed progenitors for either the AT1 or the AT2 lineage form much earlier during development than anticipated, as early as E13.5. It was also suggested using a dual transgenic approach that labeled BPs do not significantly contribute to mature pneumocytes. However, it is unclear from this work what proportion of epithelial progenitors labeled prior to E15.5 are truly unipotent.

Uncovering the mechanisms regulating alveolar lineage specification and differentiation will help refine our current models of this fundamental developmental process. For example, previous work from our lab has demonstrated a critical role for fibroblast growth factor 10 (FGF10) signalling via fibroblast growth factor receptor 2b (FGFR2b) on AT2 lineage formation. For instance, when analyzed at PN3, heterozygous *Fgf10+/-* mice showed a decreased ratio of AT2 cells over total EpCAM-positive epithelial cells compared to wildtype controls [8], suggesting that FGF10 signalling is required for AT2 lineage formation. Interestingly, after hyperoxia exposure, which leads to a reduction in the AT2 pool of wildtype lungs, the lungs of *Fgf10+/-* mice exposed to hyperoxia actually showed a normalization of the AT2/EpCAM ratio to that seen in control lungs under normoxic conditions. This raises the interesting possibility that compensatory populations of AT2 cells become activated upon lung injury [8].

Prior to the initiation of the alveolar differentiation program, FGFR2b signalling also tightly regulates branching morphogenesis [5]. Research continues on the regulatory mechanisms downstream of FGFR2b signalling which control, at the level of the multipotent epithelium, the transition from a branching program during the pseudoglandular stage (E11.5–E16.5) to an alveolar program at the canalicular/saccular stages (E16.6–E18.5). For example, it was shown that the forced activation of FGF-dependent Kras expression in the distal epithelium suppressed the alveolar differentiation program by promoting, via SOX9, the branching program [9]. This supports loss-of-function experiments which have demonstrated that SOX9 acts downstream of FGF signalling to promote epithelial branching while preventing epithelial differentiation [10]. Although overexpression of *Fgf10* from E10.5 to E12.5 did not affect epithelial fate, continuous overexpression of *Fgf10* throughout lung development (from E11.5 to E18.5) prevents the formation of SOX2-positive bronchiolar progenitor cells while maintaining a high number of SOX9-positive cells [10, 11]. Interestingly, overexpression of FGF9 from E10.5 to E12.5, which signals through epithelial FGFR3, potently suppressed differentiation of SOX9-positive progenitors to SOX2-positive epithelial cells [11]. In addition, analysis of FGF10 overexpression compared to control lungs indicated a drastically increased number of SFTPC-positive cells, suggesting that FGF10 could enhance the differentiation of the progenitor cells towards the AT2 lineage [10, 11]. FGF10 overexpression at later stages (from E15.5 to E18.5) also prevented the differentiation of already committed SOX2*-*positive bronchiolar progenitor cells to the ciliated-cell lineage but facilitated instead their commitment to the P63-positive basal-cell lineage. In these experimental conditions, both SFTPC and PDPN-positive cells were observed, but the lack of quantification prevented determining whether the overexpression of FGF10 led to changes in the number of AT1 vs. AT2 cells [10]. Loss of epithelial *Fgfr2* expression in distal airway progenitor cells using the *Id2-CreERT* driver line from E15.5 led to a decreased AT2/AT1 cell ratio at E18.5. A similar result was observed with pregnant females treated with PD0325901 (an extracellular-signal-regulated kinase (ERK) inhibitor) at E15.5 and analyzed at E18.5 for the differentiation of ID2-lineage traced cells [4]. Furthermore, the deletion of *Etv5*, a well-known FGF target gene, in adult AT2 cells resulted in the loss of AT2 markers and collateral acquisition of an AT1 signature in these cells [12]. Altogether, these results suggest that FGF plays a critical role both in the differentiation of the distal progenitors towards the AT2 fate during development as well as in the maintenance of the AT2 cell signature.

More recently, we have identified transcriptional targets of FGFR2b signalling on distal epithelial cells during early (E12.5) and mid- (E14.5) pseudoglandular development [13, 14]. In this work, we used a dominant negative transgenic mouse model to conditionally inhibit FGFR2b signalling (*ROSA26*^*rtTA/rtTA*^*; Tg(tet(o)sFgfr2b)/*+) and to identify and characterize sets of target genes. These ‘FGFR2b gene signatures’ correspond to the observed biological activities controlled by FGFR2b. During early pseudoglandular development, FGF10 acts directly on distal tip progenitors to control cell motility, adhesion and arrangement [13]. This morphogenic role persists through mid-pseudoglandular development (E14.5), along with a shifting contribution to the increased proliferation of distal cells seen at this stage [14]. A critical finding from these analyses was that loss of FGFR2b signalling led to a significant decrease in AT2 signature gene expression as early as E12.5 [11, 13]. This finding suggests that *bona fide* pneumocyte progenitors may exist earlier than currently appreciated and that their regulation is concomitant with the dominant branching program.

In the current paper, we build upon our work at E12.5 and at E14.5 by looking here at the role of FGFR2b signalling during late pseudoglandular/early canalicular stage (E16.5) development. We use Cre-based transgenic mouse models to conditionally inactivate FGFR2b signalling in AT1 or AT2 progenitors and to label those cells with a tomato-RFP reporter. With these models, we were able to lineage label putative distal airway progenitors just prior to E16.5 and analyze their commitment to both AT2 and AT1 lineages in control and loss of FGFR2b signalling conditions. We also FACS-isolated E18.5 AT1 and AT2 cells derived from labeled AT2 progenitors at E14.5-E15.5. Results from these experiments suggest that a significant proportion of alveolar epithelial progenitors labeled during mid- and late pseudoglandular development display inter-lineage commitment, and that FGFR2b signalling restricts that commitment. Furthermore, to identify potential transcriptional targets involved in this differentiation regulation, we globally inhibited FGFR2b ligand activity in E16.5 lungs for nine hours using the aforementioned dominant negative mouse model. We performed a gene array on mutant and littermate control lungs and found a set of downregulated genes. We identified, using published single-cell RNA-sequencing datasets of E17.5 and adult lung samples, cell-types expressing these FGFR2b signature genes. This data-mining approach highlights the extensive heterogeneity in broadly classified cell-types, as well as suggesting intriguing roles for FGFR2b signalling in subsets of pneumocytes during repair after injury. Taken together, our findings help build a model for the specific role of FGFR2b signalling on distal epithelial progenitors during pseudoglandular lung development, and suggest a role for FGFR2b on a subset of a generally heterogenous population of alveolar epithelial cells during lung repair after injury.

## Materials and methods

### Ethical statement and husbandry

Animal experiments and harvesting organs and tissues from wildtype and mutant mice following euthanasia using pentobarbital were approved at Justus Liebig University Giessen by the federal authorities for animal research of the Regierungspraesidium Giessen, Hessen, Germany (Approved Protocol GI 20/10 Nr. G 84/2016).

All mice used to generate experimental and control embryos were housed in a specific pathogen-free (SPF) environment with free access to food and water. Up to five females were housed together, while males were housed singly. Females between 9 and 12 weeks of age were used to generate embryos.

### In vivo mouse models

Experiments to inhibit FGFR2b ligands were conducted using a previously described and validated inducible dominant negative mouse model: *ROSA26*^*rtTA/rtTA*^*; Tg(tet(o)sFgfr2b)/*+ (B6-Cg-Gt(ROSA)26Sor^tm1.1(rtTA,EGFP)Nagy^ Tg(tetO-Fgfr2b/lgh)1.3Jaw/sbel) [13, 15]. These mice were generated by crossing *Rosa26*^*rtTA/rtTA*^ mice with *Rosa26*^*rtTA/rtTA*^; Tg(*tet(o)sFgfr2b)/*+ mice to obtain experimental (*Rosa26*^*rtTA/rtTA*^*; Tg(tet(o)sFgfr2b)/*+) and littermate control (*Rosa26*^*rtTA/rtTA*^*;* +*/*+) embryos. This model employs a reverse tetracycline transactivator (rtTA) under the transcriptional control of the ubiquitous *Rosa26* locus. Upon administration of doxycycline, the rtTA binds to the tetracycline operator (tetO), inducing the transcription of a soluble dominant negative form of *Fgfr2b* (*sFgfr2b*), which is secreted from cells and acts to sequester all FGFR2b ligands in the extracellular matrix.

Cell-autonomous *Fgfr2b* deletion and lineage labelling in alveolar progenitors was achieved by using a cell-type specific CreERT2 recombinase combined with a tandem dimer (*td*)*Tomato*^*flox*^ reporter and an *Fgfr2b*^*flox*^ knock-in line. Upon administration of tamoxifen, CreERT2 translocates to the nucleus, recombining the loxP-flanked stop cassette upstream of the *tdTomato* construct, as well as excising the IIIb exon in the *Fgfr2* gene. Targeting of AT1 progenitors was achieved by creating *Hopx*^*CreERT2/*+^*; Fgfr2b*^*flox/flox*^*; tdTomato*^*flox/flox*^ embryos (Stock *Hopx*^*tm2.1(cre/ERT2)Joe*^*Fgfr2*^*tm1Dsn*^*Gt(ROSA)26Sor*^*tm9(CAG-tdTomato)Hze*^/sbel). Experimental litters were obtained by crossing *Hopx*^*CreERT2/*+^*; Fgfr2b*^*flox/flox*^*; tdTomato*^*flox/flox*^ mice with *Hopx*^+*/*+^*; Fgfr2b*^*flox/flox*^*; tdTomato*^*flox/flox*^ mice, whereas control litters were obtained by crossing *Hopx*^*CreERT2/*+^*; Tomato*^*flox/flox*^ mice with *tdTomato*^*flox/flox*^ mice. Targeting of AT2 progenitors was achieved by creating *Sftpc*^*CreERT2/*+^*; Fgfr2b*^*flox/flox*^*; tdTomato*^*flox/flox*^ embryos (STOCK *Sftpc*^*tm1(cre/ERT2,rtTA)Hap*^*Fgfr2*^*tm1Dsn*^* Gt(ROSA)26Sor*^*tm9(CAG-tdTomato)Hze*^/sbel) [24]. Experimental litters were obtained by crossing *Sftpc*^*CreERT2/*+^*; Fgfr2b*^*flox/flox*^*; tdTomato*^*flox/flox*^ mice with *Sftpc*^+*/*+^*; Fgfr2b*^*flox/flox*^*; tdTomato*^*flox/flox*^ mice, whereas control litters were obtained by crossing *Sftpc*^*CreERT2/*+^*; tdTomato*^*flox/flox*^ mice with *tdTomato*^*flox/flox*^ mice.

Induced constitutive expression of FGFR signalling in labeled AT2 progenitors was accomplished using a *Sftpc*^*CreERT2/*+^*; Rosa26*^*rtTAflox/*+^*; Tg(tet(o)caFgfr1/*+*; tdTomato*^*flox/*+^ mouse line (STOCK *Sftpc*^*tm1(cre/ERT2,rtTA)Hap*^*Gt(ROSA)26Sor*^*tm1.1(rtTA,EGFP)Nagy*^Tg(tetO-Fgfr3*R248C/Fgfr1)#Dor Gt(ROSA)26Sor^*tm9(CAG-tdTomato)Hze*^/sbel) [16]. Pregnant females carrying littermate experimental (expressing *Tg(tet(o)caFgfr1*) and control (without *Tg(tet(o)caFgfr1*) embryos were used for experiments.

### In vivo activation of CreERT2/loxp and rtTA/tet(o) systems

Timed-pregnant females were used to conduct in vivo experiments, where the embryonic day (E) 0.5 was assumed to be noon on the day a vaginal copulation plug was found.

To activate the CreERT2/loxp system, tamoxifen, dissolved in corn oil, was administered at the desired timepoint via an intraperitoneal injection (Tam-IP) (Dosage: 0.1 mg tamoxifen/g mouse weight). To activate the rtTA/tet(o) system, doxycycline, dissolved either in PBS (for injection) or in water, was administered at the desired timepoint via an intraperitoneal injection (Dox-IP) (Dosage: 0.0015 mg doxycycline/g mouse weight), or through drinking water (Dosage: 200 µg doxycycline/ml water).

### Euthanasia

At the endpoint of an experiment, a lethal dose of pentobarbital sodium was administered to animals via IP injection (Dosage: 0.4 mg pentobarbital/g mouse weight). After breathing ceased and a lack of pupil response to light was observed, cervical dislocation was performed to ensure death. Embryos were then harvested and briefly kept in PBS on ice before lung dissection.

### Embryonic lung dissection and imaging

Shortly after embryo harvest, lungs were dissected under a stereomicroscope as detailed in Jones and Bellusci [17]. Briefly, embryos were placed in PBS in a petri dish. Tails were removed for genotyping. Working with fine-tipped forceps and dissecting scissors, lungs were very carefully and gently removed from the chest cavity to avoid any tissue damage. Lungs were positioned in the petri dish in PBS and were imaged. Whole lungs were used for FACS, left lobes were taken for RNA isolation, and right lobes were processed for paraffin embedding.

### DNA isolation and PCR

DNA was isolated from embryonic tails and genotyped following standard lab procedures. PCR products were detected using capillary gel electrophoresis. A list of gene-specific primers can be found in supplementary Table S1.

### RNA isolation and RT-qPCR

FACS-isolated AT1 and AT2 cells were put in 700 µl QIAzol Lysis Reagent (Qiagen), and lysed by vortexing. Total RNA was isolated using the miRNeasy Mini Kit (Qiagen), and eluted in 30 µl RNase-free water. RNA amount and purity were assessed using a NanoDrop 2000c spectrophotometer. Up to 1 µg of total RNA for each sample was reverse transcribed using the QuantiTect Reverse Transcription Kit (Qiagen, Hilden, Germany).

Primers were designed to amplify specific mature mRNAs using NCBI’s primer-BLAST option (https://www.ncbi.nlm.nih.gov/tools/primer-blast/) (last accessed, 07-10-2022). A list of gene-specific primers can be found in supplementary Table S2.

qPCR reaction mixtures were set up using the PowerUp SYBR Green Master Mix (Thermo Fisher, Schwerte, Germany), with a final volume of 20 µl for each reaction. Samples were run with two technical replicates on a LightCycler 480II using the following protocol: UDG activation at 50 °C for 2 min; DNA polymerase activation at 95 °C for 2 min; and 40 cycles of denaturation at 95 °C for 15 s, annealing at 60 °C for 15 s, and extension at 72 °C for 1 min. To validate amplification specificity, a dissociation step was also included for each sample. Threshold cycles (Ct) were calculated and used for relative expression analyses, using mouse *Hprt* as the reference gene.

∆*C*_t_ values were calculated according to the following formula:

$$\Delta C_{{\text{t}}} { = }C_{{{\text{t}}\;{\text{Reference}}}} - C_{{{\text{t}}\;{\text{gene of interest}}}} .$$

Unpaired two-tailed Student’s *t* tests were performed on the ∆*C*_t_ values, which can be assumed to be normally distributed. Number of ‘*n*’ and significance level is indicated either in the figures or in the figure legends.

### Microarray

Differential gene expression was investigated using microarray as previously detailed [13, 18]. Briefly, for RNA concentrations greater than 50 ng/µl, the T7-protocol was followed. In this protocol, purified total RNA was amplified and Cy3-labeled using the LIRAK kit (Agilent Technologies) following the kit instructions. Per reaction, 200 ng of total RNA was used. The Cy3-labeled aRNA was hybridized overnight to 8 × 60 K 60mer oligonucleotide spotted microarray slides (Agilent Technologies, design ID 028005).

For experiments where samples yielded less than 50 ng/µl of RNA, the SPIA-protocol was utilized. In this protocol, purified total RNA was amplified using the Ovation PicoSL WTA System V2 kit (NuGEN Technologies). Per sample, 2 µg amplified cDNA was Cy-labeled using the SureTag DNA labeling kit (Agilent Technologies). The Cy3-labeled aRNA was hybridized overnight to 8 × 60K 60mer oligonucleotide spotted microarray slides (Agilent Technologies, design ID 074809).

For each protocol, hybridization, washing and drying of the slides followed the Agilent hybridization protocol. The dried slides were scanned at 2 µm/pixel resolution using the InnoScan is900 (Innopsys). Image analysis was performed with Mapix 6.5.0 software, and calculated values for all spots were saved as GenePix results files. Stored data were evaluated using the R software (version 3.3.2) (https://www.r-project.org/) and the limma package (version 3.30.13) from BioConductor (http://bioconductor.org/packages/release/bioc/html/limma.html). Gene annotation was supplemented by NCBI gene IDs via biomaRt (last accessed 31-03-2021).

### FACS

The procedure to isolate RFP-labeled alveolar cells has been previously published in our lab [19]. In brief, after harvested lungs were dissociated in dispase and Collagenase Type IV at 37 °C for 40 min. with frequent agitation, single-cell suspensions were passed serially through 100-, 70- and 40-μm cell strainers (BD Biosciences). Red blood cells were eliminated using RBC lysis buffer (Sigma‐Aldrich), according to the manufacturer's protocol. Cells were then pelleted and resuspended in FACS buffer (0.1% sodium azide, 5% fetal calf serum (FCS), 0,05% in PBS) before being stained with antibodies: anti‐EpCAM (APC-Cy7‐conjugated, Biolegend,1:50), CD49F (APC‐conjugated, Biolegend,1:50), and anti‐PDPN (FITC‐conjugated, Biolegend, 1:20) for 20 min on ice in the dark, followed by washing. Next, cells were washed and stained with SYTOX (Invitrogen) according to the manufacturer’s instructions, to eliminate dead cells. Finally, flow cytometry and cell sorting were conducted using a FACSAria III cell sorter (BD Biosciences). Data were analyzed using FlowJo software version X (FlowJo, LLC).

Flow cytometry values were used in the figures. Significance was determined by unpaired two-tailed Student’s *t* tests. All data are presented as mean ± SEM. Values of *p* < 0.05 were considered significant. The number of independent samples (*n*) can be found in the figures.

### Immunofluorescence

Freshly dissected E18.5 right lung lobes were washed briefly in sterile PBS, then fixed in 4% PFA for 4 h at 4 °C, and then washed again in PBS (3 × 5 min). Lungs were dehydrated by successive washes in a graded ethanol series (30, 50, 70, 100, 100%) for 5 min each, and then stored in 100% ethanol at − 20 °C until further processing.

For paraffin embedding, lungs were washed in Xylol (3 × 5 min, or until clear), incubated for 1 h at 60 °C in a 1:1 Xylol/paraffin mixture, washed in pure paraffin (3 × 20 min) at 60 °C, and then stored in pure paraffin overnight at 60 °C. Lungs were then embedded in paraffin blocks and sectioned using a microtome to a thickness of 3–5 µm. Sections were placed in a 40 °C water bath for approximately 30 min, and then placed on glass slides and incubated at 37 °C overnight.

Before antibody staining, sections were first washed with gentle shaking in Xylol (3 × 10 min), and then in serial dilutions of ethanol (100, 70, 50, and 30%) for 3 min each, and finally in distilled water for 5 min. Sections were then washed with PBST (1 × PBS + 0.1% TWEEN20) (3 × 5 min). Blocking solution (1 × PBS + 3% bovine serum albumin (BSA) + 5% goat serum (GS) + 0.4% TritonX) was then added atop each section for 1 h at room temperature. Primary antibodies were added to incubation buffer (1 × PBS + 1.5% BSA + 2.5% GS + 0.2% TritonX) and samples were incubated overnight at 4 °C (anti-Hopx (Atlas antibodies HPA030180), anti-Ki67 (Invitrogen 14-5698-82), and anti-PDPN (Invitrogen 14-5381-82) were added at 1:200 dilution and anti-proS-PC (Millipore AB3786) and anti-proS-PB (Abcam AB40876) at 1:500 dilution). Following primary antibody incubation, samples were washed in PBST (3 × 5 min) and secondary antibodies were added (all at a 1:500 dilution) for 1 h at room temperature, in the dark. Samples were washed in PBST (3 × 10 min) and PBS for 5 min, with gentle shaking. Finally, ProLong Gold antifade reagent with DAPI (Invitrogen) was added to each section and covered with a glass coverslip.

### Proliferation and apoptosis

Proliferation was assessed using antibody staining against Ki67 (1:200 dilution). Due to the fact that Ki67 staining requires antigen retrieval and that antigen retrieval destroys the RFP protein, tomato RFP-positive cells were imaged just prior to the antigen retrieval step. After secondary staining, the original RFP images were used to identify the same region of the lung for re-imaging. Afterward, the two images of the region of interest were overlaid and cropped so that the corresponding cells matched perfectly. Images were then separated into individual channels and manually quantified using FIJI software.

Apoptosis was assessed on paraffin sections via the TdT-mediated dUTP Nick-End Labelling (TUNEL) assay using the DeadEnd Fluorometric TUNEL System (Promega) according to the manufacturer’s instructions. Apoptosis was not quantified because the number of apoptotic cells in each sample were too few.

### Correlating FGFR2b signatures to published scRNA-seq data

E17.5 lung scRNA-seq expression data from Frank et al. [7] was accessed (GEO GSE113320) and the AT1 and AT2 lineages were clustered and displayed on a tSNE plot using the Seurat v2.2 R package pipeline (http://satijalab.org/seurat/), similar to the original paper [7]. FGFR2b gene signature scores were calculated as previously described [20]. Subclustering of cluster 4 (mature AT2s) was performed using the updated Seurat v3. Plots were displayed using UMAP to increase clustering resolution. SCTransform from the Seurat package was used for data normalization and scaling. PCA was followed by UMAP using pc = 30. Clusters were identified using the FindClusters function, with a resolution of 0.5. For the heatmap, the top 50 genes of each subcluster were selected based on the average log_10_fold changes, and scaled, centered and normalized expression was displayed. The threshold to identify differentially expressed genes was set to log_10_fold change = 0.25.

### Correlating FGFR2b signatures to published scRNA-seq data from a bleomycin-induced lung injury study

For this analysis, the pre-processed data set published in Strunz et al. [21] was retrieved from the listed GitHub repository (https://github.com/theislab/2019_Strunz) and explored with SCANPY (v1.6.0), as previously described [22]. This study assessed the gene expression changes of murine lungs after bleomycin injury at multiple time points with Drop-seq. The whole lung data set was further used without modification, whereas the EpCAM^pos^ enriched data set with densely sampled time points (days 1–14, 21, 28, 35, 56) was subset to cell types belonging to the alveolar epithelium (AT1, AT2, Krt8 ADI, activated AT2). The principal components and knn graph (*n*_pcs = 10, *n*_neighbors = 20) were re-calculated and formed the input for the UMAP algorithm.

To quantify the enrichment of the FGFR2b signatures, a score was calculated using SCANPY’s tl.score_genes() on both the whole lung data set and the alveolar epithelium subset. The input signatures consisted of 42 genes for time point E12.5, 76 genes for E14.5 and 48 genes for E16.5. For visual inspection, the three scores were then overlaid onto the UMAP embeddings for both data sets.

### In situ hybridization expression data

To assess the expression patterns of genes in embryonic lungs, the online database ‘Genepaint’, which contains in situ hybridization data for many genes expressed in E14.5 lungs, was used (https://gp3.mpg.de/) (last accessed 01-03-2022). Each of the genes significantly downregulated in our E16.5 + 9 h experiment was assessed, and the ones which were clearly present were chosen for the figure.

### Microarray analyses

As detailed elsewhere [13, 18], mean spot signals were background corrected with an offset of 1 using the NormExp procedure on the negative control spots. The logarithms of the background-corrected values were quantile-normalized. The normalized values were then averaged for replicate spots per array. From different probes addressing the same NCBI gene ID, the probe showing the maximum average signal intensity over the samples was used in subsequent analyses. Genes were ranked for differential expression using an unpaired two-tailed Student’s *t* test on a moderated *t* statistic, and heatmaps were generated displaying genes according to descending *p* values. Gene set tests were done on the ranks of the *t* values, using the function ‘geneSetTest’ in the limma package from BioConductor. The number of independent samples (*n*) can be found in the figures. Gene sets were either user defined or, for pathway analyses, according to the KEGG database (last accessed 31-03-2021).

The data from the microarray experiments have been deposited in the NCBI's gene expression omnibus (GEO accession GSE115880).

### Manual quantification of immunostained cells

Immunostained sections from each sample (*n* = 3) were imaged at 63×. Multiple images (between 7 and 15) of distal alveolar regions were captured per section and exported as TIFF files. Images were processed and analyzed using FIJI (version 2.1.0/1.53c) [23]. First, images were separated into their component colour channels (red, green and blue). The average background value of each channel was determined and subtracted from the total signal value. Separately, red-positive and green-positive were manually labelled and counted. These fields were then overlaid and the double-positive cells were tallied.

Average cell counts were determined per sample. Unpaired two-tailed Student’s *t* tests were performed on the average values. All data are presented as mean ± SEM. Values of *p* < 0.05 were considered significant. The number of independent samples (*n*) can be found in the figures and figure legends.

## Results

### AT1 and AT2 progenitor lineage labeling suggests a level of cross-lineage contribution

**Fig. 1:**
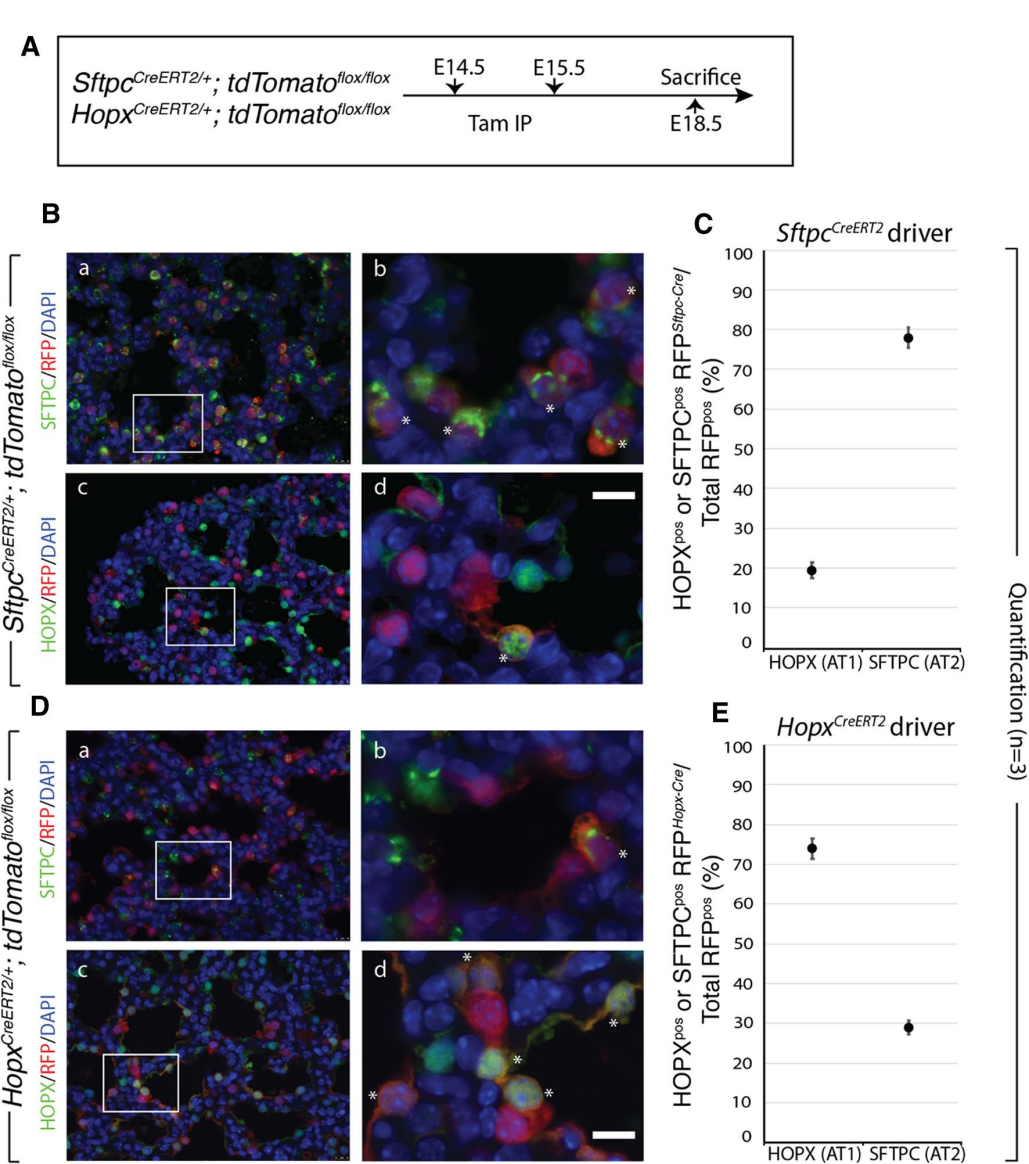
Lineage tracing of AT2 (*Sftpc*) and AT1 (*Hopx*) progenitors suggests inter-lineage contributions. **A** Transgenic mice carrying a *CreERT2* allele downstream of a *Sftpc* promoter or a *Hopx* promoter were used to label AT2 or AT1 progenitors, respectively, by recombining a floxed ‘stop’ cassette upstream of a *tdTomato* reporter. Timed-pregnant females were injected intraperitoneally with tamoxifen (Tam-IP) at E14.5 and E15.5, and embryonic lungs were harvested just prior to birth at E18.5. **B** Lineage labeled *Sftpc*^*CreERT2/*+^; *tdTomato*^*flox/flox*^ embryonic lungs were immunostained for SFTPC (a and b) and HOPX (c and d) to assess the contribution of labeled cells to the AT2 and AT1 lineages, respectively (see asterisks in panels b and d for examples of lineage labeled AT2 and AT1 cells, respectively). Scale bar: (a, c) 30 µm, (b, d) 7.5 µm. **C** Labeled AT1 and AT2 cells were counted from multiple images taken from three independent samples (*n* = 3) and are displayed as a percentage of the total number of Tomato RFP^pos^ cells. Labeled HOPX (AT1) cells compose around 19.21% ± 1.62% of the total RFP^pos^ population, whereas labeled SFTPC (AT2) cells compose around 78.09% ± 2.50%. **D** Lineage labeled *Hopx*^*CreERT2/*+^; *tdTomato*^*flox/flox*^ embryonic lungs were immunostained for SFTPC (a and b) and HOPX (c and d) to assess the contribution of labeled cells to the AT2 and AT1 lineages, respectively (see asterisks in panels b and d for examples of lineage labeled AT2 and AT1 cells, respectively). Scale bar: (a, c) 30 µm, (b, d) 7.5 µm. **E** Labeled HOPX (AT1) cells compose around 74.02% ± 1.29% of the total RFP^pos^ pool, whereas labeled SFTPC (AT2) cells compose around 29.75% ± 0.50%

It has been argued that AT1 and AT2 progenitor cell populations are largely unipotent as early as E13.5 during mouse lung development [7]. This contrasts with the earlier model proposing that the majority of mature pneumocytes pass through a bipotential progenitor well after E13.5 [6]. To address this issue, we investigated the lineage commitment of early alveolar progenitors using our mouse models. We labeled AT1 and AT2 progenitor cells during the late pseudoglandular stage using *Hopx*^*CreERT2/*+^*; tdTomato*^*flox/flox*^ (*n* = 3) and *Sftpc*^*CreERT2/*+^; *tdTomato*^*flox/flox*^ (*n* = 3) transgenic mouse lines, respectively (please note, for brevity and clarity we use slightly modified nomenclature throughout this paper. For official nomenclature, please refer to the “Materials and methods”). We labeled cells via two tamoxifen IP (Tam-IP) injections (E14.5 and E15.5) and assessed the contribution of lineage-labeled cells to each alveolar epithelial population at E18.5 (Fig. 1A). Assessing the expression of SFTPC and of HOPX proteins via immunofluorescence staining in the *Sftpc*^*CreERT2*^ line, it was observed that around 19.21% ± 1.62% and 78.09% ± 2.50% of labeled (RFP^Pos^) cells were HOPX^Pos^ and SFTPC^Pos^, respectively (Fig. 1B, C). The expression of labeled SFTPC^Pos^ and of HOPX^Pos^ cells in the *Hopx*^*CreERT2*^ line was essentially the reverse of what was found in the *Sftpc*^*CreERT2*^ line: around 74.02% ± 1.29% and 29.75% ± 0.50% of RFP^Pos^ cells were HOPX^Pos^ and SFTPC^Pos^, respectively (Fig. 1D, E).

These data demonstrate that a significant proportion of mature pneumocytes (around 20–30%) derive from lineage-flexible progenitors (both uni- and bi-potent) when labeled during mid-pseudoglandular development.

### Cell-autonomous deletion of ***Fgfr2b*** in SFTPC^pos^ AT2 progenitors pushes them toward the PDPN^pos^ AT1 lineage

To investigate the role of FGFR2b signalling specifically on AT2 progenitor cell fate, we utilized a previously validated *Sftpc*^*CreERT2/*+^; *tdTomato*^*flox/flox*^; *Fgfr2b*^*flox/flox*^ line [24]. This line labels AT2 (*Sftpc*) progenitors while simultaneously excising the IIIb isoform of the *Fgfr2* gene [25].

**Fig. 2:**
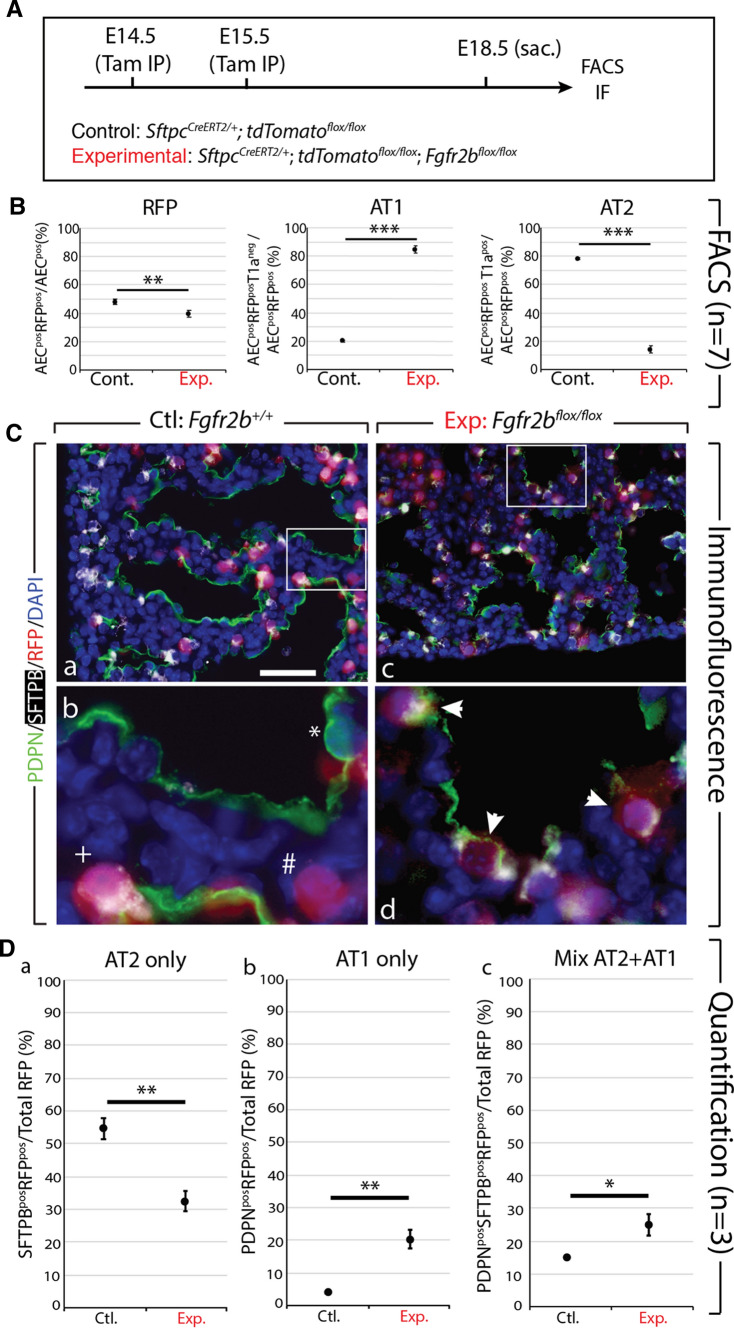
Cell autonomous deletion of *Fgfr2b* in *Sftpc*-positive progenitors results in an increased proportion of RFP-labeled AT1 cells and mixed AT1 and AT2 cells. **A** Experimental design. Pregnant females carrying either control or experimental embryos were injected with TAM-IP at E14.5 and E15.5, and embryonic lungs were harvested at E18.5. **B** Control (*n* = 7) and experimental (*n* = 7) lungs were processed for FACS analysis and isolation of cells. EpCAM^pos^ CD49f^pos^ alveolar epithelial cells (AEC) were sorted, then the tomato RFP^pos^ population was isolated. From these, AT1 and AT2 cells were isolated based on the expression of the AT1 marker T1α (Podoplanin). A decrease in the proportion of RFP-labeled alveolar epithelial cells (AEC^pos^RFP^pos^/AEC^pos^) is observed in experimental (40.6% ± 2.57%) versus control lungs (48.64% ± 1.21%). A significant increase is seen in the proportion of RFP-labeled AT1 cells (AEC^pos^ RFP^pos^ T1α^pos^/AEC^pos^ RFP^pos^) in experimental versus control lungs (85.5% ± 2.44% vs. 20.93% ± 0.64%); concomitantly, a significant decrease in labelled AT2 cells (AEC^pos^ RFP^pos^ T1α^neg^/AEC^pos^ RFP^pos^) is observed in experimental versus control lungs (14.5% ± 2.44% vs. 79.07% ± 0.64%). (*n* = 7; ***p value < 0.001). **C** Immunofluorescent staining for PDPN (AT1 marker) and SFTPB (AT2 marker) in control *Fgfr2b*^+*/*+^ (a and b) and experimental *Fgfr2b*^*flox/flox*^ (c and d) lungs. Single- and double-labeled cells are shown in ‘b’ (* = PDPN^pos^, # = RFP^pos^, + = SFTPB^pos^/RFP^pos^). Triple-labeled cells are shown in ‘d’ (arrowheads = PDPN^pos^/SFTPB^pos^/RFP^pos^). Scale bar: (a, c) 30 µm, (b, d) 7.5 µm. **D** Quantification of ‘C’ shows (a) decreased proportion of RFP-labeled AT2 (SFTPB-positive) cells in experimental versus control lungs (32.23% ± 2.94% vs. 54.34% ± 3.33%); (b) an increased proportion of RFP-labeled AT1 (PDPN^pos^) cells in experimental versus control samples (19.81% ± 2.01% vs. 4.78% ± 0.17%); (c) as well as an increased proportion of RFP-labeled cells expressing both AT1 and AT2 markers in experimental versus control lungs (24.89% ± 2.88% vs. 15.59% ± 0.13%). (*n* = 3; *p value < 0.05, **p value < 0.01)

We Tam-IP injected at E14.5 and E15.5 timed-pregnant females carrying either control (*Sftpc*^*CreERT2/*+^; *tdTomato*^*flox/flox*^) (*n* = 7) or experimental (*Sftpc*^*CreERT2/*+^; *tdTomato*^*flox/flox*^; *Fgfr2b*^*flox/flox*^) (*n* = 7) embryos and harvested the lungs at E18.5 (Fig. 2A). We then FACS-isolated alveolar epithelial cells (AEC) based on the pan-epithelial marker EpCAM and the alveolar lineage-specific marker CD49f (EpCAM^pos^CD49f^pos^). From these, RFP-labeled AT1 (AEC^pos^, RFP^Pos^, T1α^Pos^) and AT2 (AEC^Pos^, RFP^Pos^, T1α^Neg^) cells were isolated (see Fig. S1A–C for the FACS gating strategy). As Fig. 2B shows, the proportion of RFP-labeled AEC decreased from around 48.64% ± 1.21% in control samples to 40.6% ± 2.57% in experimental lungs. From these epithelial cells, the percentage of RFP^Pos^-labeled T1α^pos^ AT1 cells relative to the RFP^Pos^ epithelium (EpCAM^Pos^RFP^Pos^) drastically increased from around 20.93% ± 0.64% in controls to 85.5% ± 2.44% in experimental lungs, while the percentage of labeled AT2 (EpCAM^Pos^RFP^Pos^T1α^Neg^) cells significantly decreased from 79.09% ± 0.64% to 14.5% ± 2.44%.

To validate our FACS-isolation strategy, AT1 and AT2 gene signature expressions (as published by Treutlein et al. [6]) were assessed in isolated AT1 cells against the signature expressions in isolated AT2 cells in control lungs (*n* = 7 samples were pooled into two samples for the gene array; Fig. S1E). (Please note, due to the extreme lack of RFP^Pos^-labeled experimental AT2 cells, it was not possible to obtain enough mRNA to assess expression data on these experimental cells). As expected, the isolated AT1 cells showed an increased AT1 signature expression compared to the isolated AT2 cells, while the AT2 cells displayed an increased AT2 signature compared to the AT1 cells. qPCR results on *Fgfr2b* expression help validate our model (Fig. S1D). In the FACS-isolated RFP^pos^ AT1 cells from experimental lungs, the expression of *Fgfr2b* is drastically reduced when compared to either the control AT1 or AT2 cells.

In addition to our FACS-based analyses, we did immunofluorescent antibody staining for PDPN (an AT1 marker) and SFTPB (an AT2 marker) on RFP^pos^ control and experimental lung sections (Fig. 2D). This strategy allowed us to identify RFP-labeled cells expressing a single marker protein (either AT1 or AT2), or expressing both markers (mixed AT1 and AT2) (Fig. 2Cb, d). Quantification of these samples (Fig. 2Da–c) showed that RFP-labeled SFTPB^pos^ AT2 cells over the total RFP^pos^ pool decreased from 54.34% ± 3.33% in controls to 32.23% ± 2.94% in experimental lungs; that RFP-labeled PDPN^pos^ AT1 cells increased from 4.78% ± 0.17% in controls to 19.81% ± 2.01% in experimental lungs; and that RFP-labeled cells expressing both markers increased from 15.59% ± 0.13% in controls to 24.89% ± 2.88% in experimental samples.

Taken together, these data suggest that after cell-autonomous deletion of *Fgfr2b* in SFTPC^pos^ AT2 progenitor cells, these cells transition toward the PDPN^pos^ AT1 lineage.

### Cell-autonomous deletion of ***Fgfr2b*** in HOPX^pos^ AT1 progenitors pushes them toward the SFTPC^pos^ AT2 lineage

**Fig. 3:**
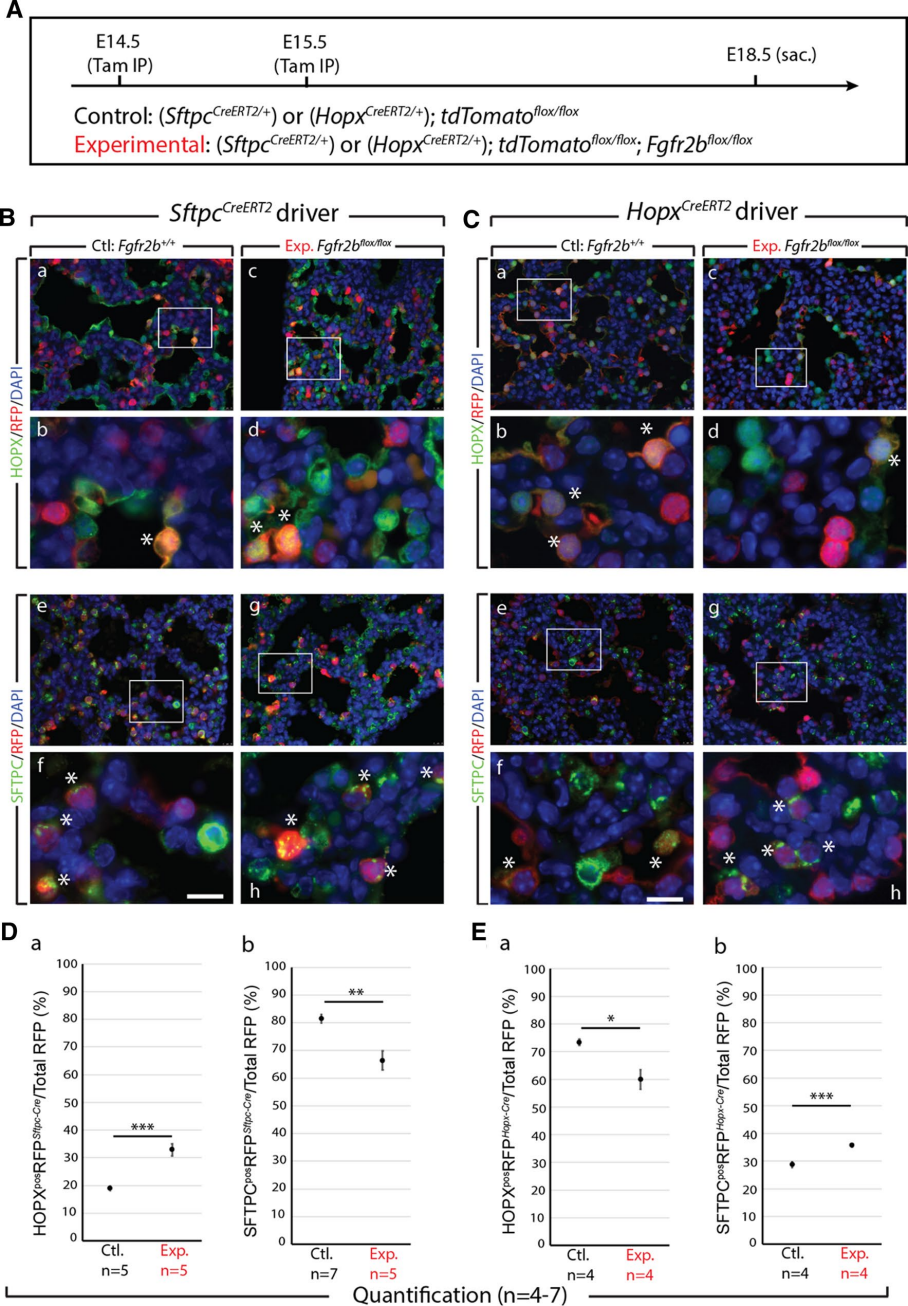
Analysis of *Fgfr2b* deletion in AT2 and in AT1 progenitors. **A** Experimental design. Pregnant females carrying either control or experimental embryos were injected with TAM-IP at E14.5 and E15.5, and embryonic lungs were harvested at E18.5. **B**, **C** Representative images of control and experimental samples from the *Sftpc*^*CreERT2*^ (B) and *Hopx*^*CreERT2*^ (C) driver lines. Immunofluorescence staining of either AT1 cells (HOPX; B and C panels a–d) or AT2 cells (SFTPC; B and C panels e–h) in control (B and C a, b and e, f) and experimental (B and C c, d and g, h) lineage-labeled (RFP) samples. Asterisks indicate double-positive cells. *Scale bar:* (a, c, e, g) 30 µm, (b, d, f, h) 7.5 µm. **D**, **E** Quantification of samples from the *Sftpc*^*CreERT2*^ driver line (D) and from the *Hopx*^*CreERT2*^ driver line (E). In the *Sftpc-CreERT2* driver line (D), (a) the percentage of HOPX^pos^ lineage-labeled RFP^pos^ cells over the total number of RFP^pos^ cells increases in experimental versus control lungs (33.25% ± 2.08% vs. 19.99% ± 0.89%); (b) whereas the percentage of lineage-labeled SFTPC^pos^ cells decreases in experimental versus control samples (66.57% ± 3.32% vs. 80.98% ± 1.49%). In the *Hopx-CreERT2* driver line (E), (a) the percentage of HOPX^pos^ lineage-labeled RFP^pos^ cells over the total number of RFP^pos^ cells decreases in experimental versus control lungs (60.37% ± 3.35% vs. 77.38% ± 0.98%); (b) while the percentage of RFP-labeled SFTPC^pos^ cells increases in experimental versus control samples (36.05% ± 0.52% vs. 28.84% ± 0.96%). (*n* = 4–7; *p value < 0.05, **p value < 0.01, ***p value < 0.001)

To further explore the lineage-flexibility of AT2 progenitors, and to address whether a similar result was observed in AT1 progenitors, we repeated the experiments using the *Sftpc*^*CreERT2*^ driver line. In addition, to label AT1 progenitors, we used a *Hopx*^*CreERT2*^ driver crossed with *tdTomato*^*flox/flox*^ mice to obtain control embryos, and crossed with *Fgfr2b*^*flox/flox*^*; tdTomato*^*flox/flox*^ mice to generate experimental embryos. We assessed cells in control and experimental lungs labeled between E14.5 and E15.5 by immunofluorescence at E18.5 (Fig. 3A). Immunofluorescence antibody staining was used to identify HOPX (Fig. 3Ba–d and Ca-d) and SFTPC (Fig. 3Be–h and Ce-h) cells in control (*n* = 3) and experimental (*n* = 3) lungs for each driver line. Counting lineage-labeled cells showed that for the *Sftpc*^*CreERT2*^ driver line, the percentage of double-positive AT1 cells (HOPX^Pos^ RFP^Sftpc−Cre^(read: HOPX-positive cells RFP lineage-labeled from the SFTPC driver)) over the total pool of RFP lineage-labeled cells significantly increased from around 19.99% ± 0.89% to 33.25% ± 2.08% in experimental lungs (Fig. 3Da); whereas the level of labeled AT2 cells (SFTPC^Pos^ RFP^Sftpc−Cre^) decreased from around 80.98% ± 1.49% to 66.57% ± 3.32% in experimental lungs (Fig. 3Db).

For the *Hopx*^*CreERT2*^ driver line, the percentage of labeled AT1 cells (HOPX^Pos^ RFP^Hopx−Cre^) from the total RFP pool tended decreased from 77.38% ± 0.98% to 60.37% ± 3.35% in experimental lungs (Fig. 3Ea), whereas a significant increase in labeled AT2 cells over total labeled cells (SFTPC^Pos^ RFP^Hopx−Cre^/RFP^Pos^) was observed (from 28.84% ± 0.96% in controls to 36.05% ± 0.52% in experimental samples) (Fig. 3Eb).

The results found for both driver lines were independent of proliferative or apoptotic effects (Figs. S2 and S3), as staining for either Ki67 (Figs. S2B and S3B) or TUNEL (data not shown) on lineage-labeled cells, respectively, revealed no changes in expression between control and experimental groups (Figs. S2C and S3C).

In addition to these loss-of-function experiments, we made use of a gain-of-function mouse model (*Sftpc*^*CreERT2/*+^*; rtTA*^*flox/*+^*; Tg(tet(o)caFgfr1); tdTomato*^*flox/*+^) in which expression of a constitutively active FGFR can be induced in AT2 cells with doxycycline (Fig. S4A) [16]. This allowed us to assess whether continuous activation of FGFR signalling would suppress the commitment of labeled AT2 progenitors to the AT1 lineage. Quantification of RFP lineage-labeled cells immuno-stained for either HOPX or SFTPC (Fig. S4B and C) revealed a significant decrease from 17.5% ± 1.27% to 11.92% ± 1.31% in the percentage of lineage labeled AT2 progenitors committing to the AT1 lineage in experimental lungs (Fig. S4Ca); whereas the percentage of lineage-labeled SFTPC progenitors tended to increase from 82.99% ± 1.27% in controls to 87.36% ± 0.86% in experimental lungs (Fig. S4Cb).

To summarize, our data suggest that a significant proportion of RFP-labeled SFTPC^pos^ AT2 or HOPX^pos^ AT1 progenitors preferentially switch lineages after cell-autonomous loss of FGFR2b signalling. Therefore, the decision of AT2 or AT1 progenitor cells to commit to the opposing lineage is partially regulated by FGFR2b signalling. In particular, FGFR2b signalling actively restricts the commitment of lineage-flexible alveolar epithelial progenitors to the opposing lineage.

### Global inhibition of FGFR2b ligands reveals a set of potential direct targets of FGFR2b signalling at E16.5

**Fig. 4:**
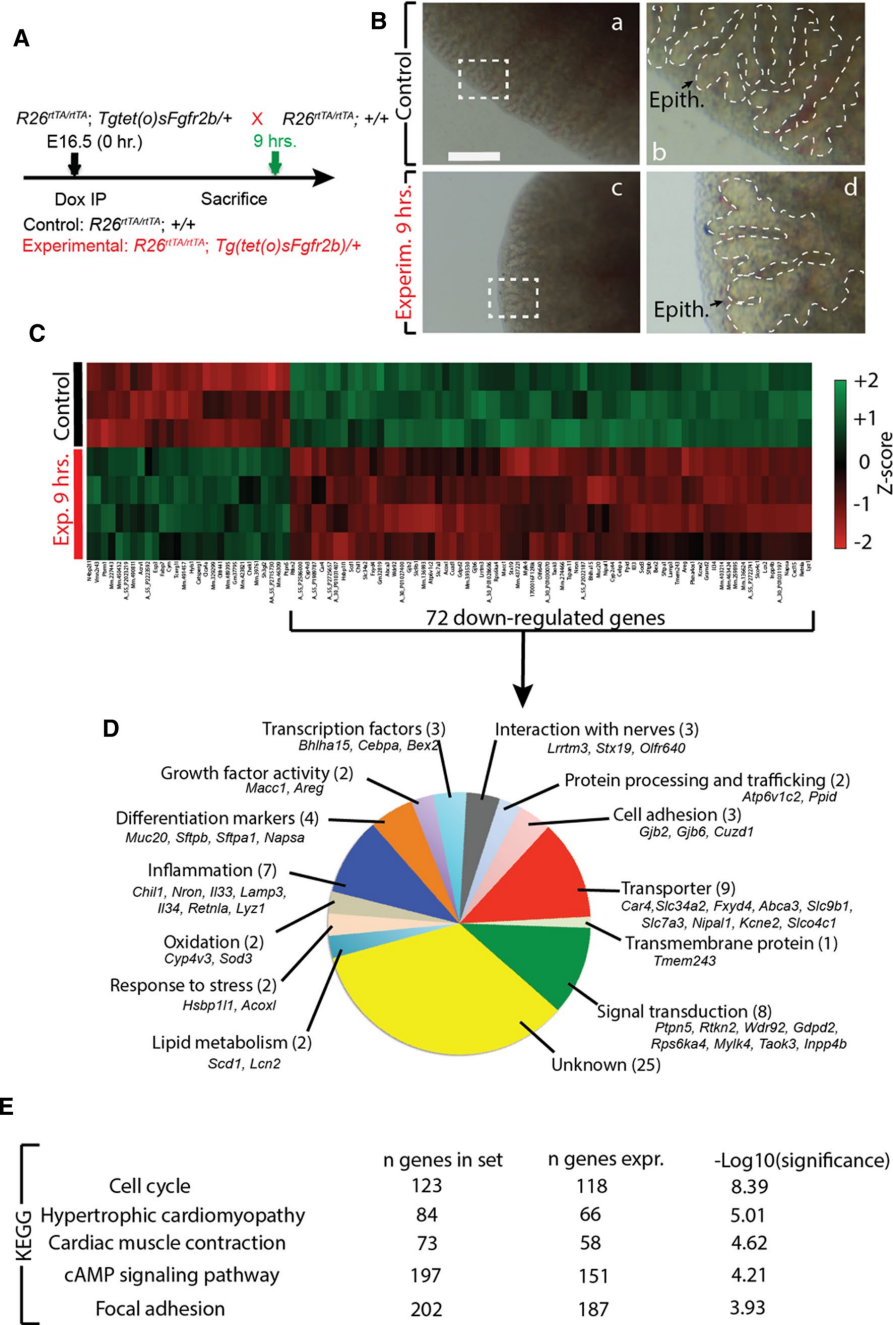
Transcriptomic changes upon FGFR2b signalling inhibition at E16.5 + 9 h. **A** Experimental design. E16.5 littermate experimental and control lungs were collected 9 h after a single Dox-IP injection. **B** Gross airway morphology (hashed line traces the epithelium) shows airway tip dilation between control (a and b) and experimental (c and d) lungs. (Epith. = Epithelium). Scale bar: (a, c) 500 µm, (b, d) 125 µm. **C** Heatmap of the top 100 genes (according to *p* value) differentially expressed after 9 h FGFR2b inhibition (*n* = 3 control and 4 experimental). 72 of those genes are down-regulated, representing potential direct targets of FGFR2b signalling. **D** Gene ontology of the 72 down-regulated genes found in ‘D’. **E** Top five KEGG pathways regulated from the complete gene array

To identify potential targets of FGFR2b signalling regulating the differentiation of distal airway progenitor cells we took a global approach to inihibit FGFR2b signalling at the protein level on the distal epithelium at E16.5. We utilized a previously described and validated transgenic mouse model: *Rosa26*^*rtTA/rtTA*^; *Tg(Tet(o)sFgfr2b)/*+ [13–15, 18]. In this system, a soluble form of the FGFR2b receptor controlled by a Tet(o) promoter (*Tg(Tet(o)sFgfr2b)*) is transcribed upon activation by doxycycline of the ubiquitously expressed rtTA (*Rosa26*^*rtTA*^). This system is rapidly activated, allowing for the fast and efficient sequestering of all FGFR2b ligands in the lung. Here, we injected E16.5 timed-pregnant females carrying experimental (*Rosa26*^*rtTA/rtTA*^; *Tg(Tet(o)sFgfr2b)/*+) and littermate controls (*Rosa26*^*rtTA/rtTA*^; +*/*+) with doxycycline intraperitoneally (Dox-IP) and harvested the embryos and their lungs nine hours later (Fig. 4A). In our previous studies, this early timepoint allowed the identification of well-accepted FGFR2b transcriptional targets at E12.5 and at E14.5 [13, 14, 26].

Macroscopically, the phenotypes of control and experimental lungs were quite similar, although the tips of the experimental buds showed the characteristic dilation of the distal lumens using this inhibitory mouse model (compare Fig. 4Bd with Bb) (see [13]). A gene array was conducted on whole lung homogenates from control (*n* = 3) and experimental (*n* = 4) embryos. A heatmap of the top 100 genes (according to *p* value) differentially regulated between experimental and control samples is shown in Fig. 4C. From these 100 genes, 72 were down-regulated in experimental versus control lungs; these genes (of which only 13 can currently and reliably be classified as epithelial specific) constitute the FGFR2b signature at E16.5 (see Fig. S5). Gene ontology (Fig. 4D) and KEGG pathway analyses (Fig. 4E) of these 72 genes indicate the biological processes regulated by FGFR2b signalling at this time point. These include, in order from high to low of the number of genes involved, cellular transporting (9 genes), signal transduction (8), inflammation (7), markers of differentiation (4), transcriptional regulation (3), cell adhesion (3), interaction with nerves (3), protein processing and trafficking (2), lipid metabolism (2), response to stress (2), oxidation (2), growth factor activity (2), and transmembrane proteins (1). The pathways primarily affected by these genes include cell cycle regulation, cAMP signalling, focal adhesion, and, interestingly, hypertrophic cardiomyopathy and cardiac muscle contraction, which have been associated with the migration of AT2 progenitors out of the lumen to escape the physical forces applied by the amniotic fluid, a process regulated by FGFR2b-mediated actin/myosin-dependent mechanisms [4].

### FGFR2b-responsive genes during pseudoglandular development narrow on a subpopulation of AT2 cells at E17.5

The 72 genes identified in this study, together with the signatures found at E12.5 and E14.5, provide a valuable set of candidate transcriptional targets involved in FGFR2b signalling (See Fig. S6 for the gene signatures at each time point). A shortcoming of this global approach, however, is the inability to accurately characterize and assess the relative contribution to the global response from the different epithelial cell types.

One way to tackle this lack of information is to data-mine published single-cell RNA-sequencing (scRNA-Seq) datasets at different developmental time points to obtain gene expression data for genes of interest. In this vein, we mined the recently published scRNA-Seq data from Frank et al. [7], which contains transcriptome data from 7106 individual *Nkx2-1*-positive epithelial cells obtained from E17.5 mouse lungs (GEO accession code GSE113320). Of the 11 categories denoted by the authors, four are of interest to us in this paper: AT1 precursor (preAT1), AT1, AT2 precursor (preAT2), and AT2 [7]. These groups are depicted and colour-coded in Supplementary Figure S7A, along with the expressions of the AT2 markers *Sftpc* and *Lamp3,* and the AT1 markers *Ager* and *Aqp5* (Fig. S7B), as well as the expression of *Fgfr2* and the well-established downstream effector of FGFR2b signalling, *Etv5* (Fig. S7C). Note the comparable expressions of *Fgfr2* in preAT2 and AT2 cells, but also the presence of this gene in preAT1 cells. Finally, we assessed the expression in the four clusters of the signature gene sets we discovered at E12.5 [13] and at E14.5 [14] (Fig. S6), and at E16.5 (this paper) (Fig. S7D–G). As the gene sets approach the time point of the sequenced single cells (E17.5), the expression profile becomes increasingly concentrated in a subset of the preAT2 and AT2 clusters (Fig. S3G). This finding suggests that the FGFR2b signalling target genes discovered using our global FGFR2b ligand inhibition model at E16.5 are predominantly expressed by a subset of the AT2 lineage at E17.5.

**Fig. 5:**
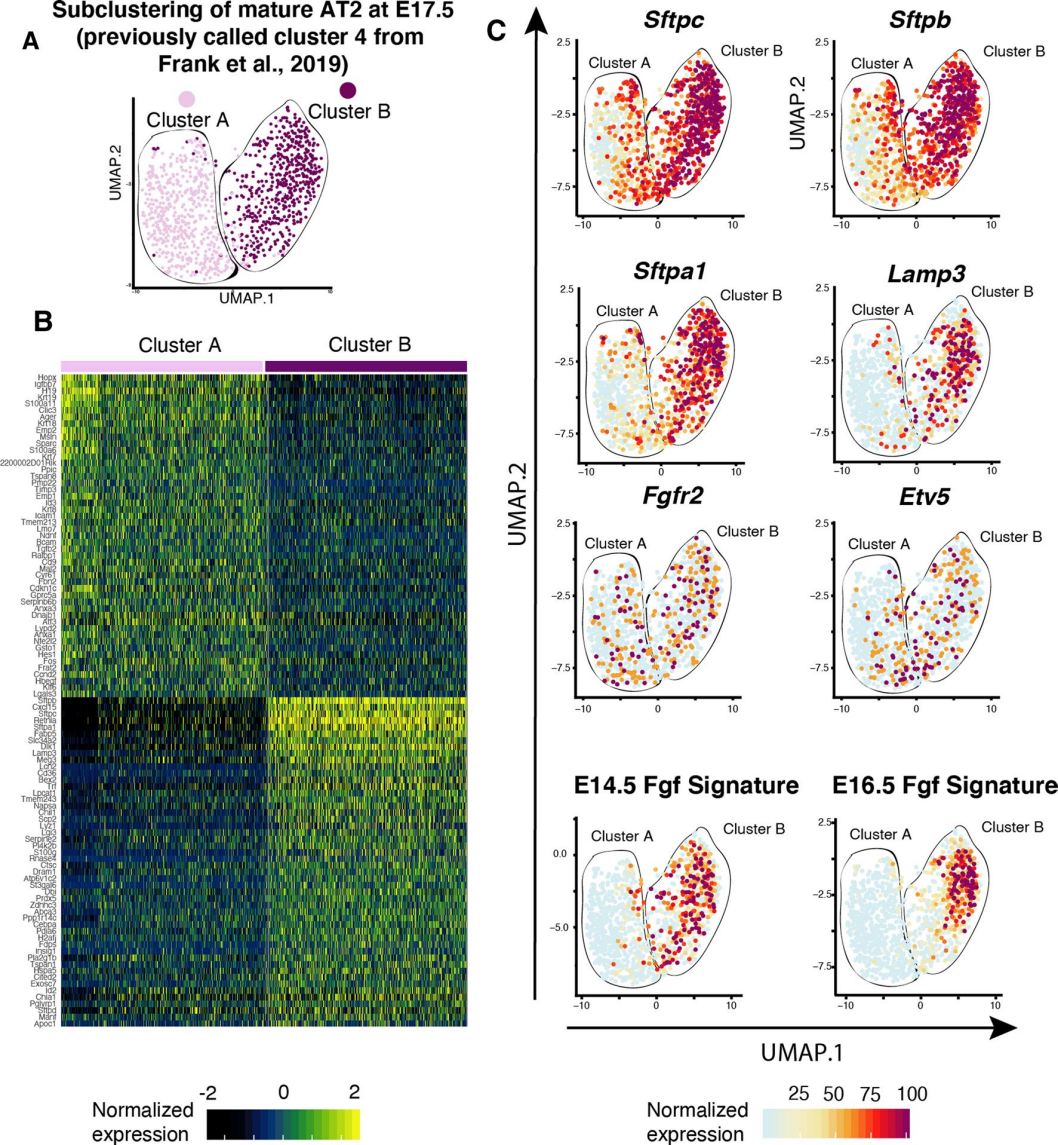
FGFR2b signature concentrates on a subcluster of mature AT2 cells at E17.5. **A** Subclustering of mature AT2 cells as identified by scRNA-seq at E17.5 [7] (GEO accession code GSE113320). The cells cluster into two discrete subclusters, termed A and B. **B** Heatmap showing the 50 upregulated genes in each subcluster, according to Log_10_fold change. While subcluster B is enriched in canonical AT2 marker genes, subcluster A shows enrichment in AT1 markers (see Fig. S8). **C** The expression profiles of select genes in subclusters A and B show high expression of markers for mature AT2 cells in cluster B: *Sftpc, Sftpb, Sftpa1* and *Lamp3*. Note how *Fgfr2b* and *Etv5*, markers for FGFR2b signalling, are scattered throughout subclusters A and B, while the FGFR2b signalling signatures determined by our lab at E14.5 and at E16.5 concentrate on a subcluster of cluster B

Following the identification of a subset of cells within the AT2 lineage that highly express the FGFR2b signature at E16.5, we reanalyzed the mature AT2 cluster (cluster 4 from Frank et al. [7]). We found that this cluster groups into two subclusters, which we have termed cluster A and cluster B (Fig. 5A). We created a heatmap showing the top 50 differentially upregulated genes in each cluster (according to average Log_10_fold change) (Fig. 5B). While canonical markers of AT2 cells, such as *Sftpc*, *Sftpb*, *Sftpa1*, and *Lamp3* are expressed in each subcluster, they are more highly expressed in cluster B (Fig. 5C), suggesting that cluster B might represent a more mature pool of AT2 cells. Interestingly, the genes most highly expressed in cluster A are predominately markers of the AT1 lineage. Indeed, assessing the expression of the AT1 and AT2 signature genes published by Treutlein et al. [6] in clusters A and B reveals a stark contrast between the two clusters: cluster A is highly enriched in AT1 marker genes, whereas cluster B expresses the AT2 signature (Fig. S8A and B). In fact, a closer look at the AT2 signature in cluster B reveals that this cluster is composed of at least two more subgroups defined by signature gene-expressing cells. Finally, while *Fgfr2* and *Etv5* expressions are scattered somewhat uniformly throughout clusters A and B (Fig. 5C), the targets of FGFR2b signalling from E14.5 to E16.5 concentrate ever more narrowly in a subcluster of cluster B. The characterization of this FGFR2b-responding subcluster remains to be determined. One hypothesis is that cluster A or B might contain the recently published ‘injury-activated alveolar progenitors (IAAPs)’ [19], albeit at an early stage of development. Figure S8C and D provide some preliminary data suggesting the equivalent of activated IAAPs might be found in the subcluster of cluster B, which also contains the aforementioned *bona fide* AT2 signature and FGFR2b signature activity.

In summary, using published scRNA-seq data from E17.5 mouse lungs, we were able to identify subclusters of cells based on FGFR2b signature genes over pseudoglandular development. These subclusters emerge from previously designated ‘mature AT2s’ [7], which, upon further subgrouping, can be divided into two major subclusters. One of those subclusters displays AT1-like gene expression patterns, while the other seems to contain *bona fide* AT2s. Within the AT2 cluster, an additional subcluster exists which is high in FGFR2b-responding cells, and to a lesser extent, activated IAAPs. These data highlight the extreme heterogeneity inherent in the alveolar cell lineages, and the issues such heterogeneity raises in classifying these cells.

### AT2 cells lose FGFR2b signalling as they transition to AT1 during repair after injury in the adult lung

A recent publication assessed the cell-type-specific transcriptomic sequences involved in the regeneration of the airway after bleomycin-induced injury [21]. Using a combination of longitudinal scRNA-seq data (GEO accession code GSE141259), RNA trajectory modeling in pseudotime, as well as lineage tracing experiments, the authors demonstrated that during lung regeneration, populations of the airway and AT2 stem cells converged on a transitional cell-type characterized by high *keratin 8* (*Krt8*) expression. These *Krt8*-positive airway differentiation intermediate (*Krt8*^Pos^ ADI) cells eventually gave rise to AT1 cells during the repair of the gas exchange surface. Since FGFR2b signalling plays a critical role in the differentiation of pneumocytes during normal development, it is also likely involved during repair after injury. Therefore, we asked what is the level and where is the expression of the FGFR2b signatures previously found at E12.5, E14.5, and in this paper at E16.5, in adult lung cells following bleomycin injury using this published scRNA-seq dataset.

**Fig. 6:**
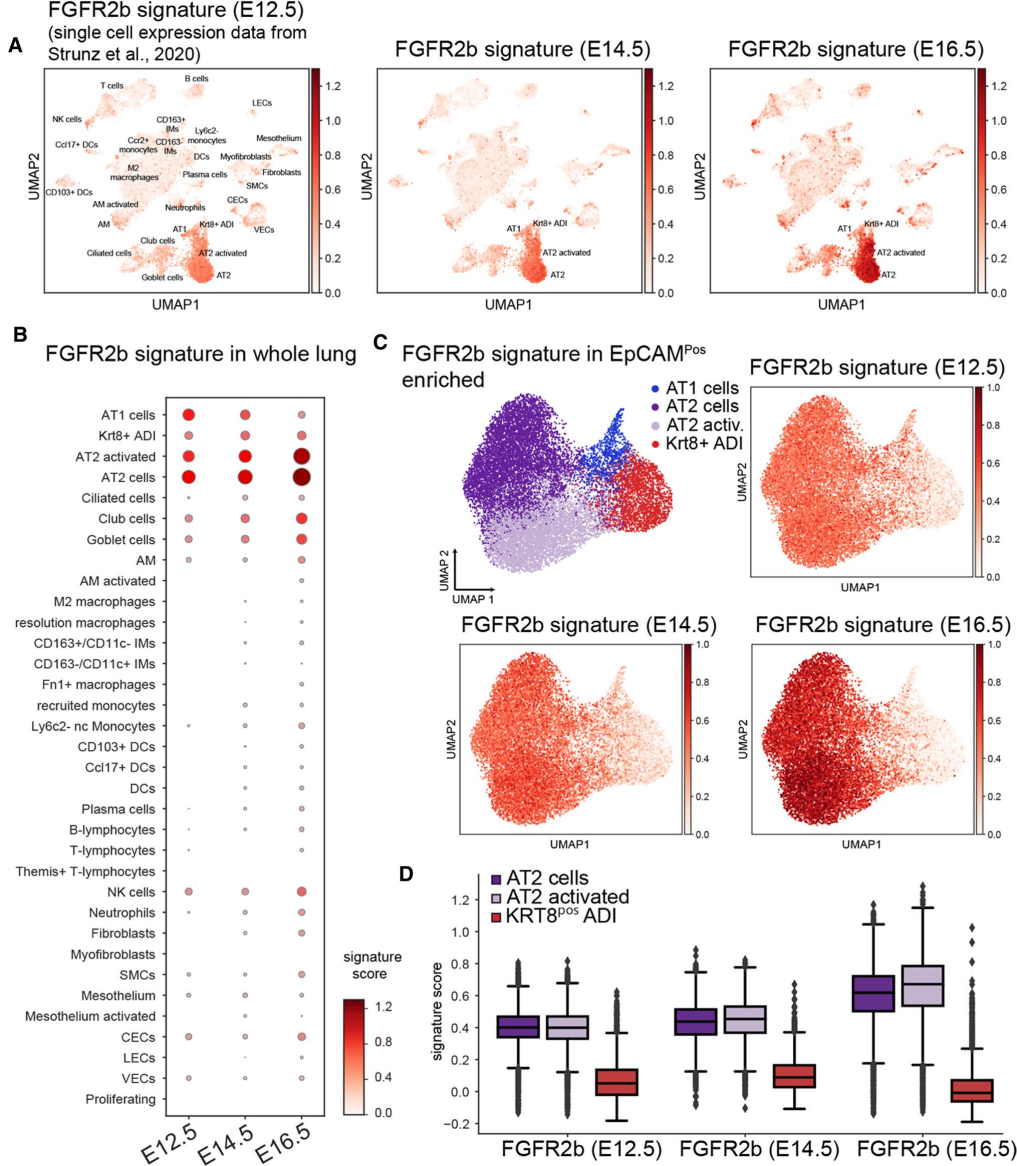
FGFR2b signalling is lost in AT2 cell transition to AT1 during repair after injury. **A** scRNA-seq data from Strunz et al. [21] (GEO accession code GSE141259) reanalyzed using the FGFR2b gene signatures found by our lab at E12.5, E14.5, and E16.5. Note the increasing expression of these signature genes as embryonic development progresses in the AT2 clusters. **B** Dot plot showing the different cell categories from ‘A’ and the relative FGFR2b signature scores at each embryonic timepoint in each category. **C** The FGFR2b signature from different embryonic time points (E12.5, E14.5 and E16.5) concentrate in the AT2 clusters of Epcam^pos^ enriched alveolar cells. **D** Box and whisker plots of the AT2 clusters, along with the KRT8^pos^ ADI cluster found in ‘C’ showing the FGFR2b signature score at the given time points. While each embryonic FGFR2b signature remains relatively high in the AT2 cells, each is virtually non-existent in the transitional KRT8^pos^ ADI cluster

Figure 6A reproduces the UMAP from the original Strunz et al. [21] study and highlights the FGFR2b signatures we found at E12.5, E14.5, and E16.5. As can be seen, the signatures from each embryonic developmental stage increasingly concentrate on the AT2 and AT2-activated cell populations found during homeostasis and during repair after bleomycin-induced injury (Fig. 6B, C). Note that the E12.5 signature is nearly equally present in AT1, AT2, and AT2-activated cells (left column in Fig. 6B). As development progresses through E16.5, FGFR2b signalling concentrates on the AT2 and AT2-activated populations (Fig. 6C), with a minor scattering of expression in KRT8^Pos^ ADI cells and mature AT1 cells. Indeed, as AT2 stem cells transition through a KRT8^Pos^ ADI state towards an AT1 fate, FGFR2b signature gene expression is significantly decreased (Fig. 6D and Fig. S9). These findings suggest that FGFR2b signalling acts on the AT2 pool during homeostasis but is lost in AT2 cells transitioning to the AT1 pool during repair after injury. Therefore, in the adult lung, as during development, FGFR2b signalling might actively prevent AT2 cells from transitioning to an AT1 fate during homeostasis.

## Discussion

**Fig. 7:**
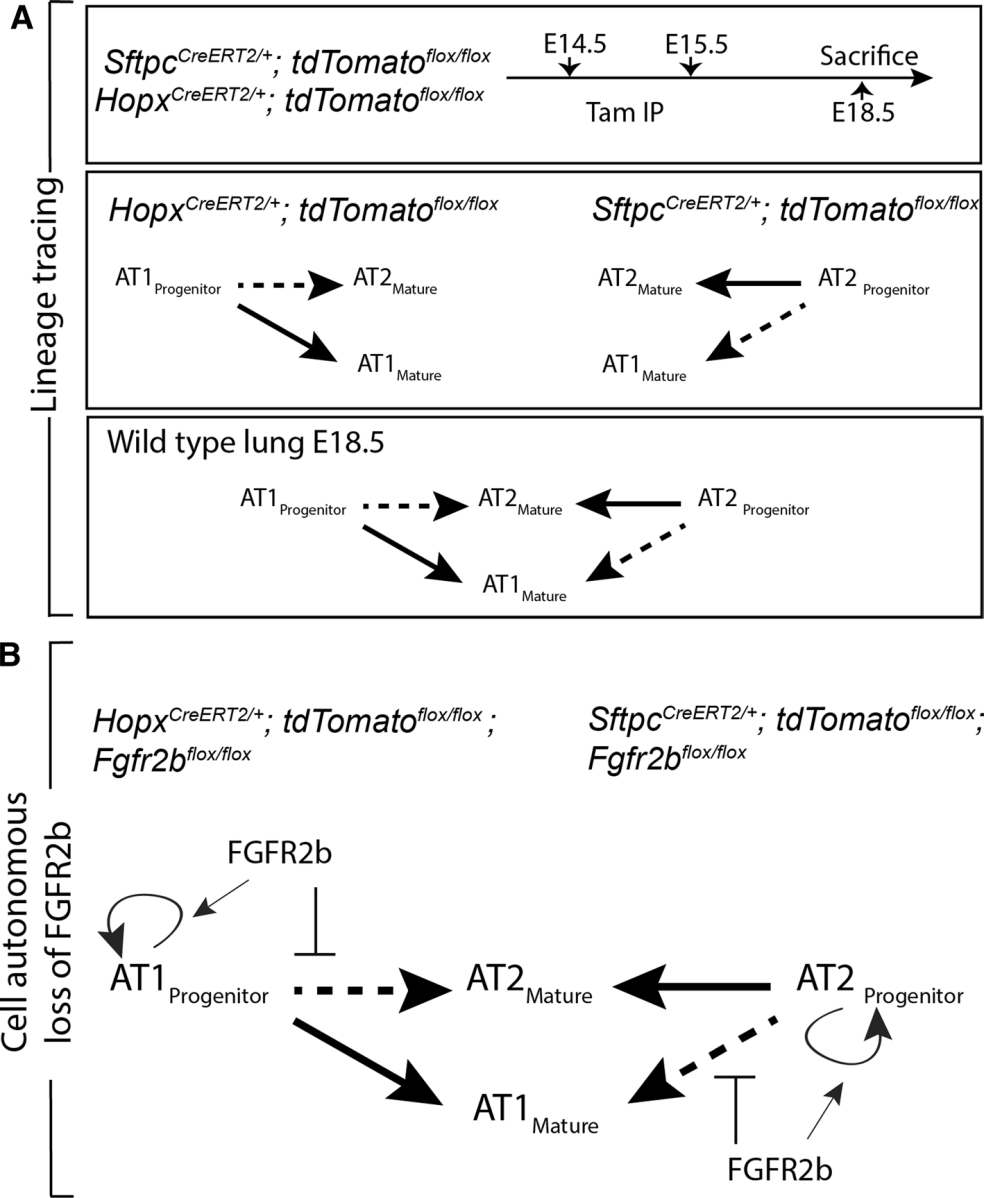
Summary and model of FGFR2b regulation of alveolar epithelial lineage formation. **A** Lineage tracing experiments at E14.5 and E15.5 (late pseudoglandular/early canalicular) embryonic development (first panel) showed that labeled AT1 progenitors give rise primarily to mature AT1s at E18.5 (solid arrow), but do contribute a significant minority to the AT2 lineage (dashed arrow), while the reverse is true for labeled AT2 progenitors (second panel). Taking the lineage tracing data together, we propose the model in the third panel of alveolar epithelial progenitor contribution to mature alveolar epithelial cells in wild-type lungs at E18.5. **B** Cell-autonomous deletion of *Fgfr2b* experiments suggest that, apart from the expected impacts of FGFR2b signalling on alveolar epithelial progenitor proliferation, FGFR2b *prevents* alveolar epithelial progenitors from actively transdifferentiating to the opposing lineage

In this paper, we have used mouse models to label distal epithelial progenitor cells at E14.5 and E15.5, and assess the contribution of these cells to mature alveolar epithelial cells at E18.5, both in wildtype lungs and in the context of FGFR2b signalling. We found that both AT1 and AT2 progenitors show a level of inter-lineage flexibility, in that labeled progenitors will give rise to the opposing lineage at E18.5. This was shown to be controlled in part by FGFR2b signalling. In the case of AT2 progenitors, loss of *Fgfr2b* led to an increase in the commitment of labeled cells to the AT1 lineage. This was supported by gain-of-function of FGFR signalling, whereby less cells committed to the opposing lineage. In the case of AT1 progenitors, the opposing results were observed; loss of *Fgfr2b* in labeled AT1 progenitors led to an increase in committed AT2 cells. Thus, FGFR2b signalling appears to maintain the stability of both the committed AT1 and AT2 cell lineages during late pseudoglandular stage lung development. These results are summarized in the model in Fig. 7. Furthermore, to obtain a set of potential FGFR2b targets regulating early progenitor differentiation, we used a global approach to inhibit FGFR2b signalling in E16.5 lungs. From this, we identified a set of downregulated genes which we termed the ‘E16.5 FGFR2b signature’. This signature, along with the signatures we had previously found at E12.5 and at E14.5, was used to data-mine published scRNA-seq datasets. We found that FGFR2b-responsive genes cluster at E17.5 into a subcategory of previously classified ‘mature AT2s’. Furthermore, we found that in a model of lung repair after injury in adult mice, the FGFR2b signature genes are effectively lost in AT2 cells transdifferentiating to AT1 cells. This finding supports the evidence found during development that FGFR2b signalling prevents AT2 cells from differentiating to AT1s.

### Cross contribution of AT1 and AT2 progenitors to the opposite lineages

The relative contribution of the early respiratory progenitors to the AT1 and AT2 lineages remains controversial. What proportion of mature pneumocytes pass through a bipotential progenitor, for example? Our results suggest that a significant proportion (between 20 and 30%) of committed (at E18.5) AT1 and AT2 cells derive from lineage-flexible progenitors expressing an opposing cell marker. This calls into doubt the model of early lineage specification, which posits that the vast majority of distal airway cells are lineage committed as early as E13.5 [7]. Furthermore, the paper from Frank et al. [7] shows that around 63% of dual-traced (HOPX^pos^ SFTPC^pos^) progenitors give rise to mature AT1 or AT2 cells at P0. Due to the few cells actually labeled in their dual-lineage tracing model (which might be from a lack of labeling efficiency), these bipotent progenitors represented a minority of the total progenitors labeled at E15.5. Were these bipotent progenitors labeled at E13.5, perhaps the evidence would suggest a much larger contribution to the mature pools of distal airway cells. More work, therefore, needs to be conducted to accurately determine the relative ratios of progenitor types and their eventual fates during pseudoglandular stage development.

### FGFR2b signalling on alveolar epithelial lineage formation and maintenance

Research on the role of FGFR2b signalling on distal airway cells has largely looked at AT2 cells in the context of adult homeostasis and repair after injury. For example, using the bleomycin-induced lung fibrosis model, it was reported that mice with specific deletion of *Fgfr2* in SFTPC-positive AT2 cells were less able to repair after injury, showed increased mortality, and had fewer AT2 cells overall [27]. This study also showed that even in the absence of injury, *Fgfr2b* deletion resulted in enlarged airspaces and increased collagen deposition, as well as a decrease in the number of AT2 cells, suggesting that FGFR2 signalling is required for AT2 maintenance.

These results support earlier work that looked at the loss of *Etv5* in AT2 cells during homeostasis and repair after injury [12]. Here, it was shown that ETV5 was required to maintain AT2 cells, for in its absence, AT2 cells transdifferentiated to AT1s. Furthermore, loss of *Etv5* in AT2 cells drastically impaired the repair process of the epithelium after lung injury, resulting in fewer AT2 cells altogether. It is well established that ETV5 is regulated by FGFR2b signalling [26, 28], and it was suggested that ETV5 protein stability in AT2 cells is controlled by Ras-mediated Erk signalling [12].

Recent work proposes the idea that during homeostasis and repair after injury FGFR2b signalling maintains AT2 cell identity independent of proliferative effects [29]. In addition, this paper also found that loss of FGFR2 signalling in AT2 cells led to their transdifferentiation to AT1s, which supports what we have found during development. Another study looking at the role of FGFR2 signalling during late development (E16.5) similarly reports that FGFR2 was required to maintain AT2 cell fate and to prevent transdifferentiation to the AT1 lineage [30]. In this paper, the authors, like us, argue against the early lineage specification model, suggesting, instead, that early alveolar progenitors remain largely uncommitted up until E16.5.

Our work remains unique in specifically studying the role of FGFR2b signalling on distal epithelial progenitors throughout pseudoglandular development [13, 14]. Unsurprisingly, we have found that FGFR2b signalling clearly affects AT2 lineage formation. As early as E12.5, for example, just after nine hours FGFR2b ligand inhibition or *Fgfr2* conditional inactivation, the AT2 signature found in distal tip progenitors was significantly decreased [11, 13]. However, as the current paper argues, this is only part of the story. Our data strongly suggests that FGFR2b signalling not only directly affects the AT2 lineage but directly the AT1 lineage as well. The cell-autonomous loss of *Fgfr2b* in labeled SFTPC^Pos^ AT2 progenitors or in HOPX^Pos^ AT1 progenitors leads to an increase in the ratio of labeled cells belonging to the alternate lineage (Figs. 4, 5). These findings are independent of possible differential proliferative or apoptotic effects (Figs. S3 and S5), and therefore represent impacts on cellular differentiation.

In summary, FGFR2b signalling, while acting primarily on the AT2 lineage at later stages of development, does appear to maintain the lineage commitment of both AT2 and AT1 progenitors during late pseudoglandular and early saccular lung development (see model in Fig. 7B).

### FGFR2b-responsive cells highlight the heterogeneity of the AT2 population

Our data indicate that as distal respiratory lineages emerge and develop (beginning as early as E12.5), the role of FGFR2b signalling shifts, eventually concentrating in a sub-population of the AT2 lineage (Fig. 5). This highlights an increasingly appreciated fact that cellular populations are heterogeneous, and points to the need to identify and further classify these sub-populations of cells [19, 31, 32]. Indeed, recent work from our lab has characterized two major sub-populations of AT2 cells in adult mice: those expressing high levels of *Sftpc* and *Fgfr2b*, and those expressing low levels of both markers in addition to high expression of a cell surface protein called PD-L1 [19]. It was shown that this latter immature sub-population of AT2s is quiescent during homeostasis, but becomes activated during compensatory growth after pneumonectomy, eventually expanding to replenish the mature AT2 population. These cells were termed ‘injury activated alveolar progenitors’ (IAAPs), and were also found to be enriched in precision-cut lung slices of human fibrotic samples [33], as well as in the context of *Fgfr2b* deletion in SFTPC^Pos^ cells during homeostasis in the mouse [24]. The engagement of this quiescent population in response to injury likely depends on FGFR2b signalling. In line with this idea, the evidence presented in the current study suggests that AT2 cells can be sub-classified according to responsiveness to FGFR2b signalling. Whether this responsiveness is a consequence of geographic proximity to zones of active FGFR2b ligand activity, or whether a subset of cells is no longer capable at a molecular level to respond to FGFR2b ligands, is yet to be determined. What is evident from the current study, however, is that a subset of AT2 cells, which is activated after injury to replenish the AT1 population, highly expresses E16.5 FGFR2b signature genes (Fig. 6). By mechanisms still unknown, as these activated AT2s transition to AT1s through a KRT8^Pos^ airway differentiation intermediate state, the FGFR2b signature is effectively shut off.

In conclusion, we have presented evidence to support a model of alveolar lineage specification and differentiation which highlights the lineage-flexibility of these airway progenitors. The decision of progenitors to commit to a particular lineage is in part regulated by FGFR2b signalling, which is also likely instructive for the transdifferentiation of progenitors in the adult lung during repair after injury. We also have made a case, given the extensive heterogeneity present in currently accepted broad classifications, for a more nuanced designation of alveolar epithelial cell types.

## Supplementary Information

Below is the link to the electronic supplementary material.

Supplementary file1 (DOCX 9002 KB)

## Data Availability

Gene array data have been deposited in GEO (accession number GSE115880).
