# Supplementary material for: FGFR2b signalling restricts lineage-flexible alveolar progenitors during mouse lung development and converges in mature alveolar type 2 cells
Source: Cell Mol Life Sci. 2022 Nov 29;79(12):609. doi: 10.1007/s00018-022-04626-2 (PMC9708820; doi:10.1007/s00018-022-04626-2)
Supplement: Supplementary file 1 — Supplementary file1 (DOCX 9002 KB) [file 18_2022_4626_MOESM1_ESM.docx]

**Supplementary figures**


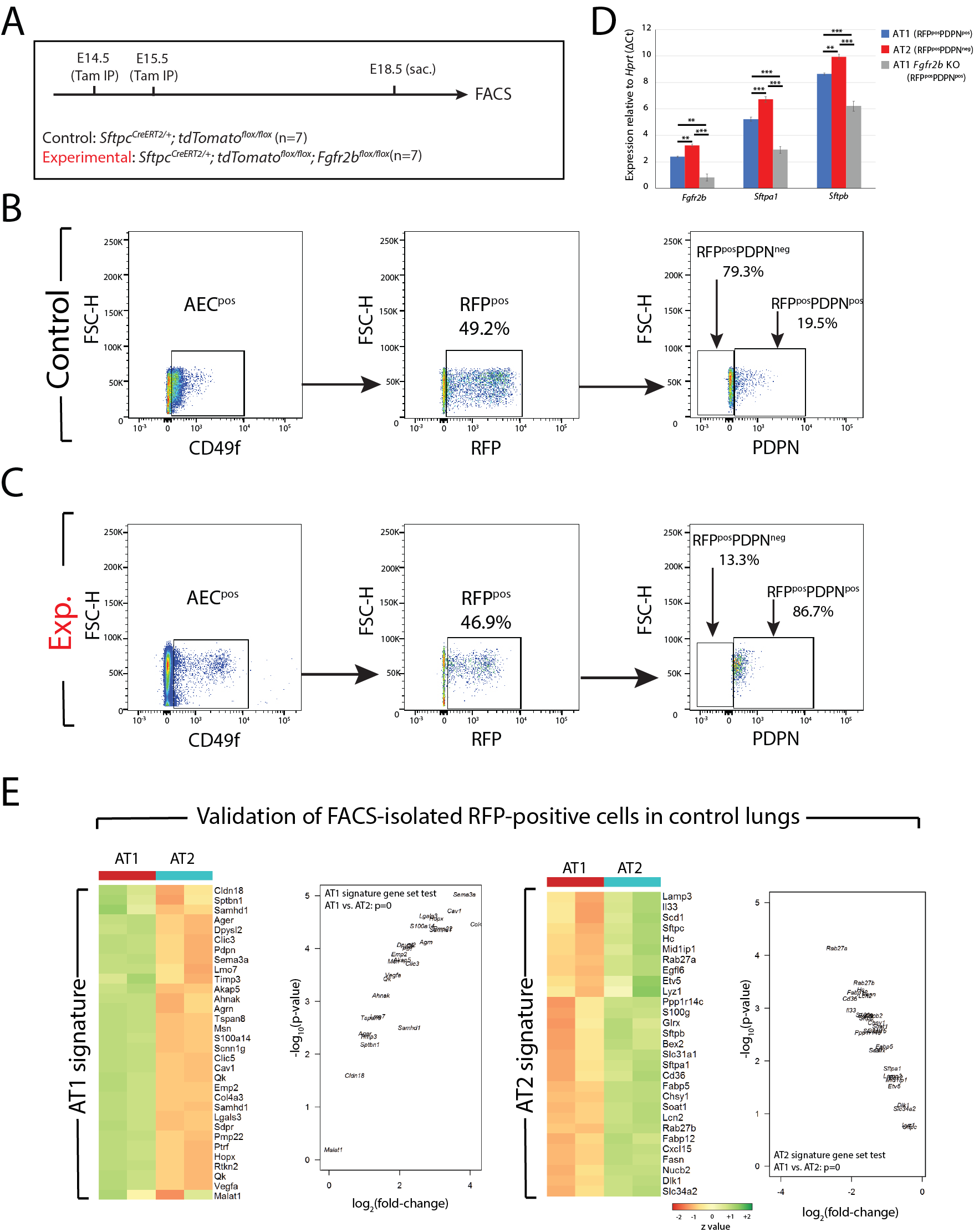


**Figure S1: FACS-isolated RFP-labeled AT1 and AT2 cells in *Sftpc^CreERT2^* driver lines shows an increase in labeled AT1 cells in experimental (*Fgfr2b^flox/flox^*) lungs. (A)** Experimental design. Pregnant females carrying control and littermate experimental embryos were Tam-IP injected at E14.5 and at E15.5. Lungs were harvested at E18.5 and prepared for FACS. **(B and C)** FACS gating strategy to isolate RFP-labeled AT1 and AT2 cells. First, EpCAM^pos^ CD49f^pos^ cells were captured. These correspond to the alveolar epithelial cell (AEC) population. Then RFP^pos^ cells were isolated from RFP^neg^ cells, and finally, PDPN^pos^ cells, corresponding to the AT1^pos^ pool, were isolated from the PDPN^neg^ population representing the AT2^pos^ pool. **(D)** qPCR data showing the expressions of *Fgfr2b*, *Sftpa1*, and *Sftpb* relative to *Hprt* in FACS-isolated control AT1 and AT2 cells (blue and red bars, respectively) and in isolated experimental AT1 cells (grey bars). The significant reduction of *Fgfr2b* expression in experimental RFP-labeled AT1 cells confirms that our model to knock-out *Fgfr2b* expression works. Note also that *Fgfr2b* mRNA is expressed in both control AT1 and AT2 cells. (n=4; *Fgfr2b* ΔCt in control AT1 is 2.37 ± 0.05, in control AT2 is 3.24 ± 0.10, and in experimental AT1 is 0.81 ± 0.26; *Sftpa1* ΔCt in control AT1 is 5.21 ± 0.16, in control AT2 is 6.72 ± 0.21, and in experimental AT1 is 2.91 ± 0.25; *Sftpb* ΔCt in control AT1 is 8.64 ± 0.07, in control AT2 is 9.94 ± 0.18, and in experimental AT1 is 6.21 ± 0.37. **p-value < 0.01, ***p-value < 0.001). **(E)** Heatmaps and volcano plots showing AT1 and AT2 signature gene expressions in FACS-isolated RFP-labeled AT1 and AT2 cells from control lungs (pooled from n=7 samples). Gene-set tests confirm that isolated AT1 and AT2 cells are highly enriched in their canonical signature genes.

**
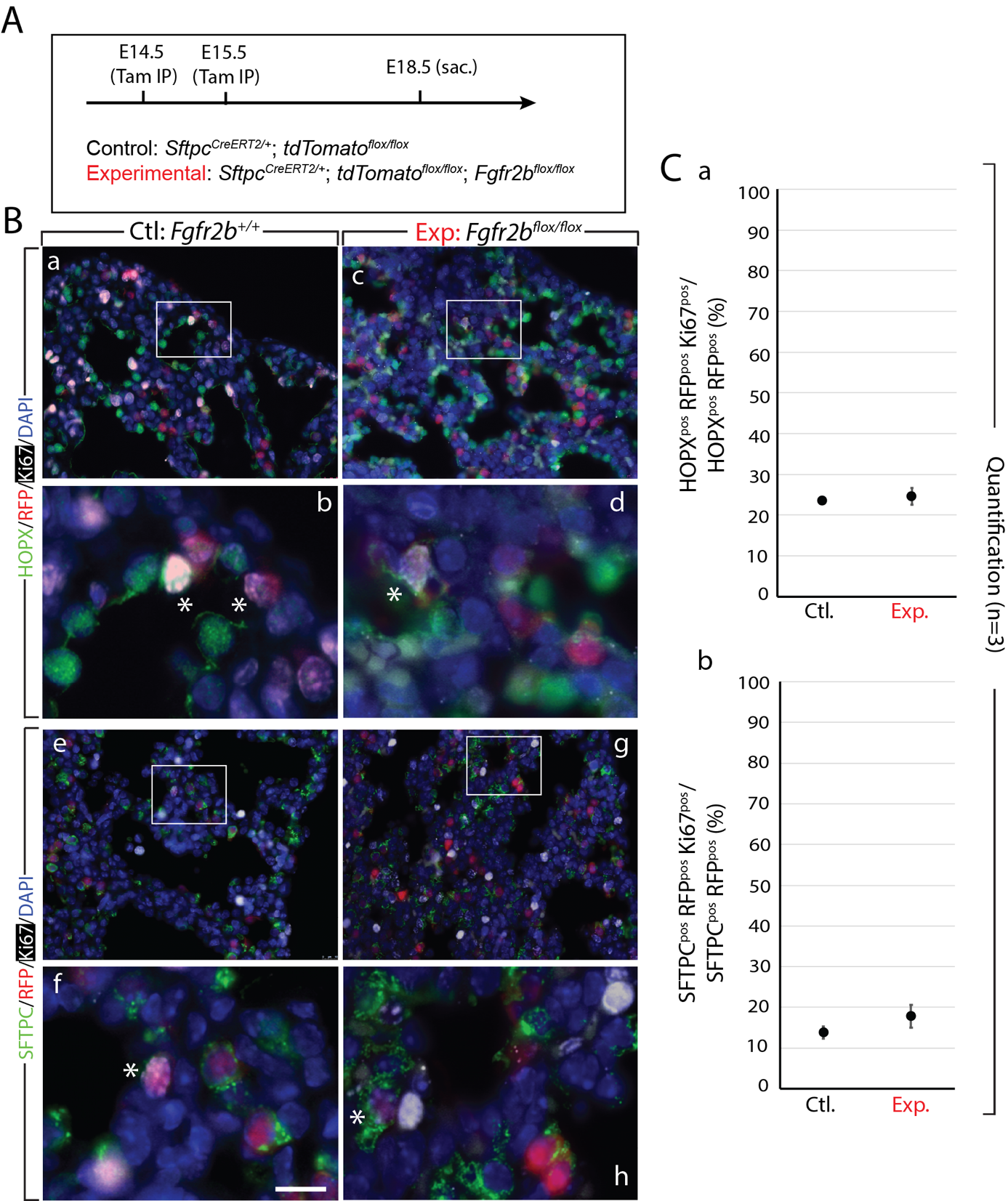
**

**Figure S2: Proliferation in RFP-labeled AT2 cells. (A)** Experimental design. Timed-pregnant females carrying control and experimental animals were Tam-IP injected at E14.5 and at E15.5 and then sacrificed at E18.5. **(B)** Proliferation was assessed by labeling samples with Ki67. HOPX^pos^ (a-d) or SFTPC^pos^ (e-h) lineage-labeled RFP^pos^ Ki67^pos^ cells were manually quantified and divided by the total HOPX^pos^ or SFTPC^pos^ RFP^pos^ pool to determine the percentage of proliferating lineage-labeled alveolar cell. Asterisks depict triple positive cells (HOPX or SFTPC, RFP, and Ki67). *Scale bar:* (a, c, e, g) 30 µm, (b, d, f, h) 7.5 µm. **(C)** Graphs show no significant difference in proliferating lineage-labeled HOPX^pos^ cells (a) (control: 23.9% ± 0.28%; experimental: 25.92% ± 2.19%) or SFTPC^pos^ cells (b) (control: 13.62% ± 1.53%; experimental: 17.75% ± 2.82%) between control and experimental groups (n=3).


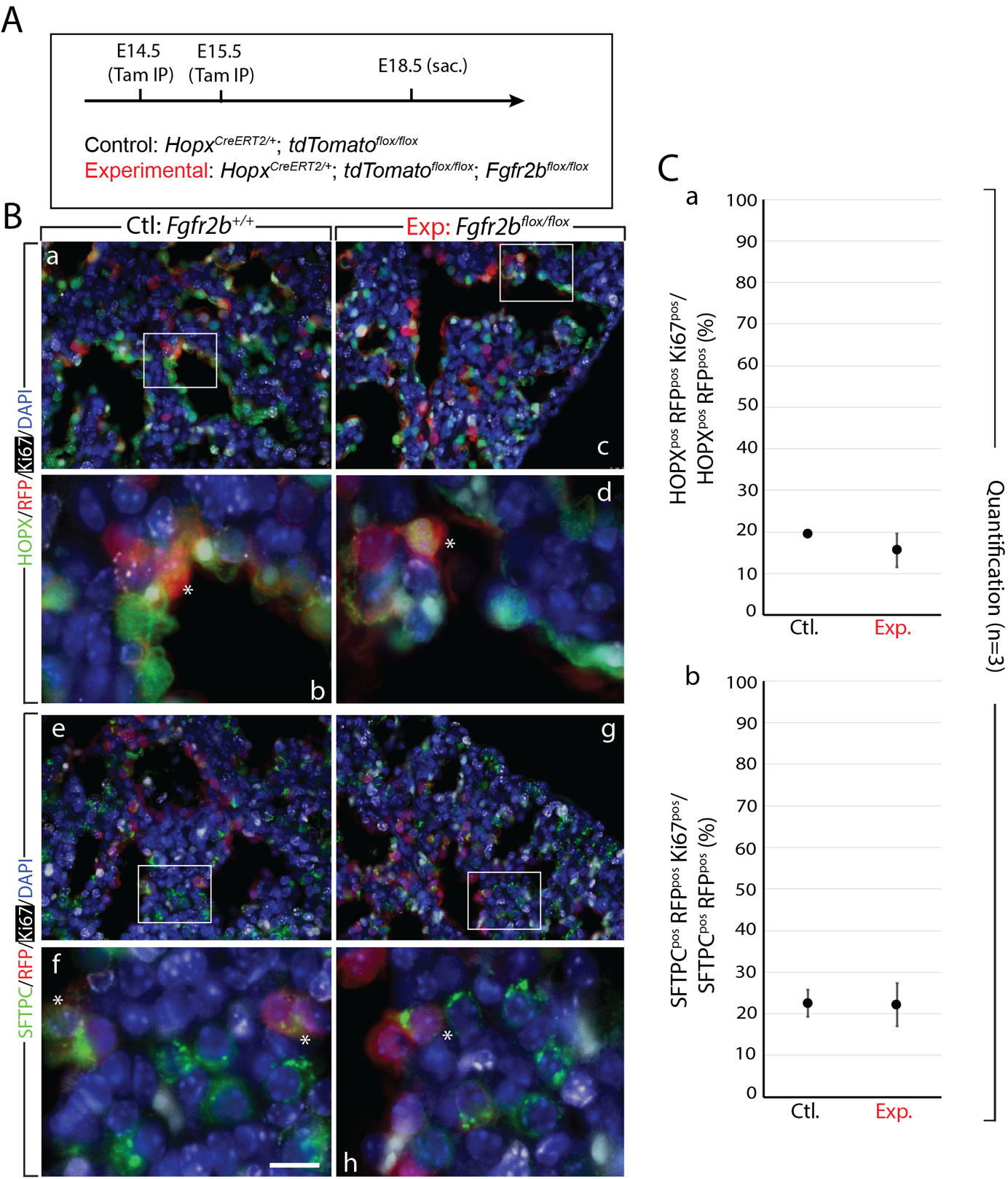


**Figure S3: Proliferation in RFP-labeled AT1 cells. (A)** Experimental design. Timed-pregnant females carrying control and experimental animals were Tam-IP injected at E14.5 and at E15.5 and then sacrificed at E18.5. **(B)** Proliferation was assessed by labeling samples with Ki67. HOPX^pos^ (a-d) or SFTPC^pos^ (e-h) lineage-labeled RFP^pos^ Ki67^pos^ cells were manually quantified and divided by the total HOPX^pos^ or SFTPC^pos^ RFP^pos^ pool to quantify the percentage of proliferating lineage-labeled alveolar cell. Asterisks depict triple positive cells (HOPX or SFTPC, RFP, and Ki67). *Scale bar:* (a, c, e, g) 30 µm, (b, d, f, h) 7.5 µm. **(C)** Graphs show no significant difference in proliferating lineage-labeled HOPX^pos^ cells (a) (control: 19.96% ± 0.56%; experimental: 15.61% ± 3.95%) or SFTPC^pos^ cells (b) (control: 22.56% ± 3.21%; experimental: 22.16% ± 5.22%) between control and experimental groups (n=3).

**
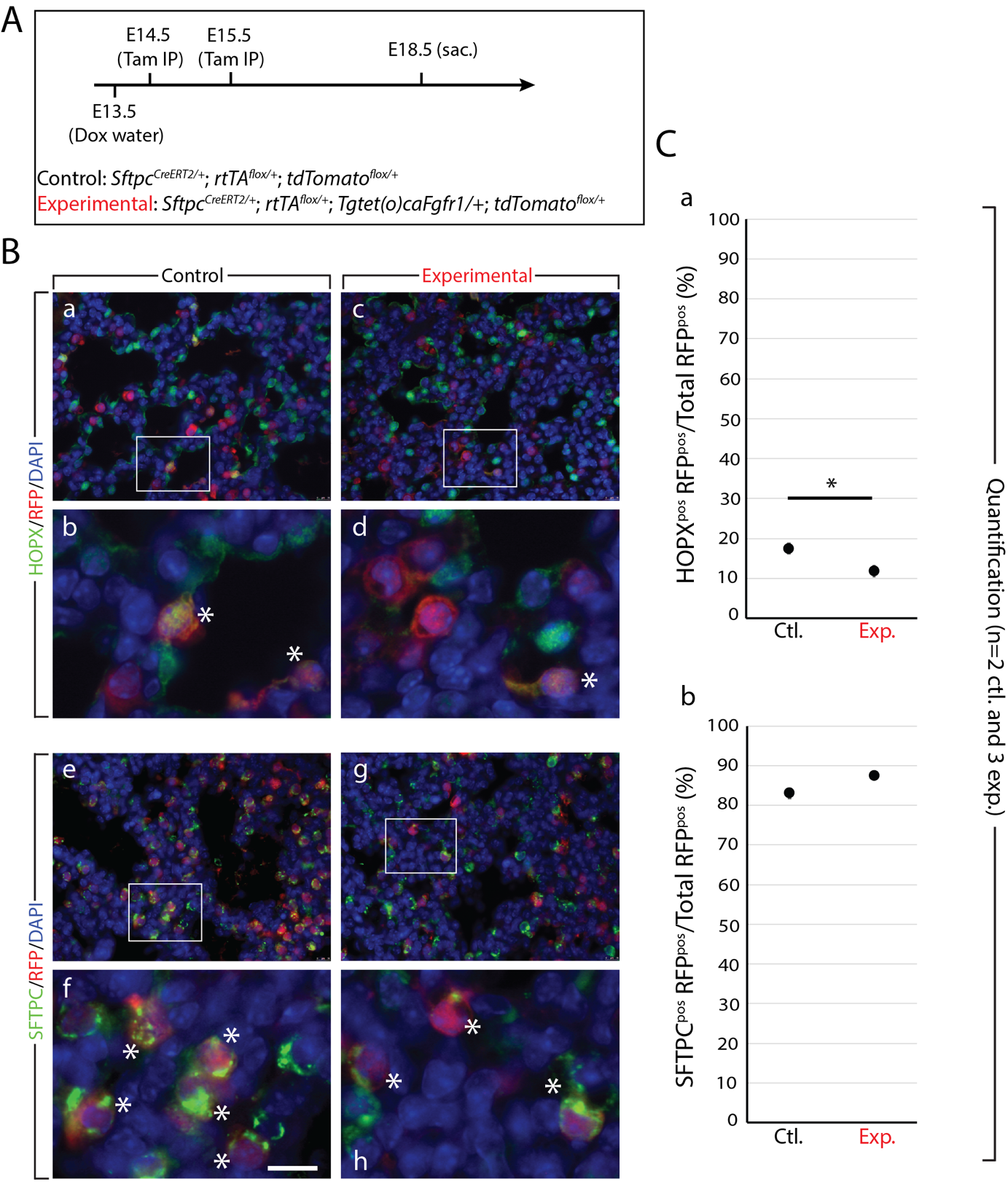
**

**Figure S4: Constitutive expression of FGFR1 in AT2 progenitors limits transition to AT1 cell fate. (A)** Experimental design. Timed-pregnant females carrying control (*Sftpc^CreERT2/+^; rtTA^flox/+^; Tomato^flox/+^*) and experimental (*Sftpc^CreERT2/+^; rtTA^flox/+^; Tg(tet(o)caFgfr1/+); Tomato^flox/+^*) embryos were fed doxycycline water from E13.5 onward. At E14.5 and at E15.5, females were Tam-IP injected. After tamoxifen injection, Cre-based recombination of the floxed *TdTomato* reporter, as well as the floxed *rtTA*, was achieved in AT2 progenitor cells. In experimental embryos, rtTA/doxycycline induces the expression of a constitutively active form of *Fgfr1*, which, when expressed in alveolar epithelial cells, mimics the activity of FGFR2b signalling. Embryos were sacrificed and lungs harvested at E18.5. **(B)** Immunofluorescence staining of either AT1 cells (HOPX; panels a-d) or AT2 cells (SFTPC; panels e-h) in control (a, b and e, f) and experimental (c, d and g, h) lineage-labeled (RFP) samples. Asterisks indicate double positive cells. *Scale bar:* 30 µm (a, c, e, g); 7.5 µm (b, d, f, h). **(C)** Quantification of samples from (B) (n=2 control and 3 experimental). (a) Graph showing the percentage of HOPX^pos^ lineage-labeled RFP^pos^ cells over total RFP^pos^ cells. There is a significant decrease from around 17.5% ± 1.27% to 11.92% ± 1.31% in these cells after constitutive FGFR1 activity. (b) Graph showing a slight upward trend in the percentage of SFTPC^pos^ lineage-labeled RFP^pos^ cells over the total of RFP^pos^ cells (control: 82.99% ± 1.27%; experimental: 87.36% ± 0.86%). (*p-value < 0.05).

**
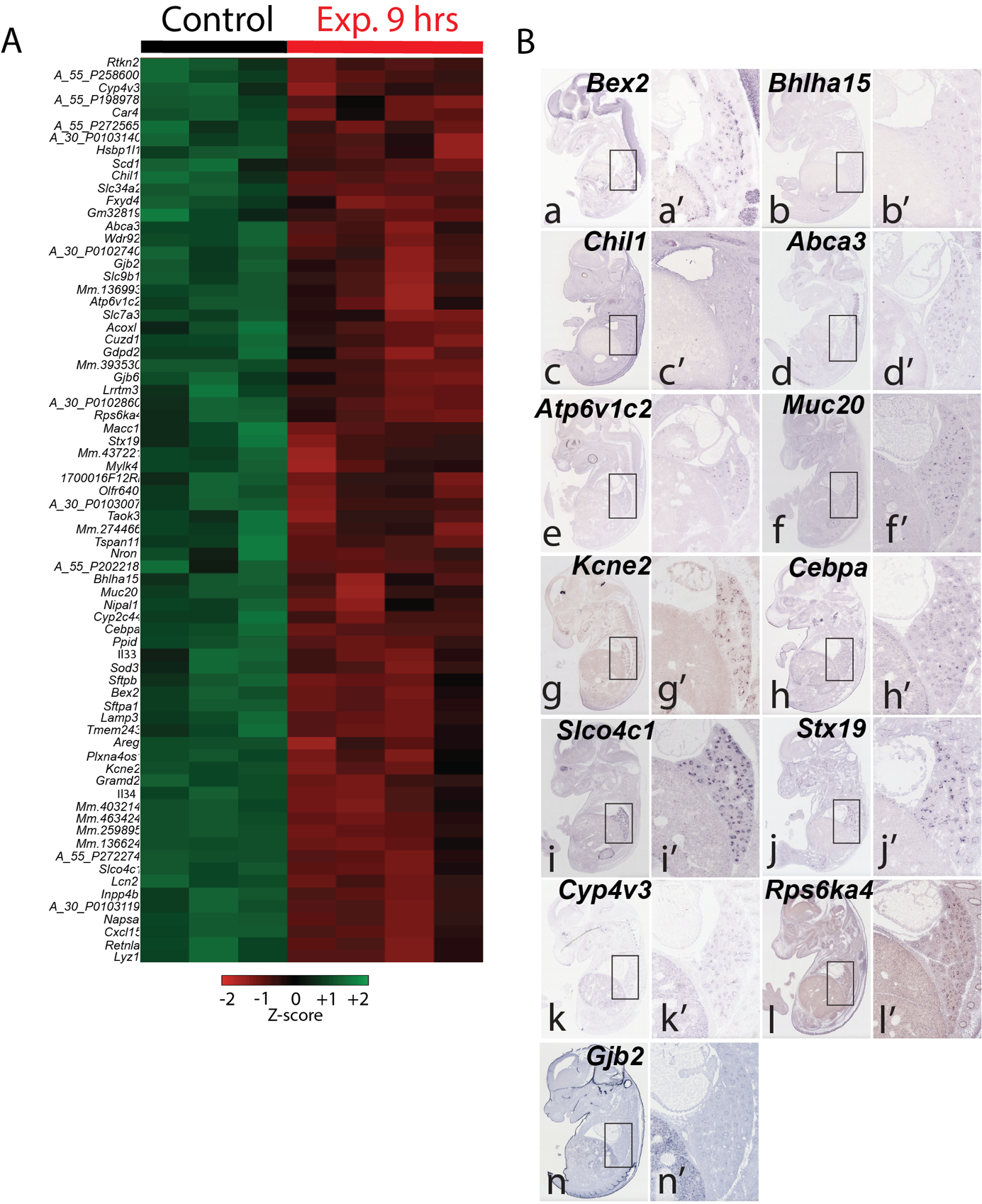
**

**Figure S5: FGFR2b signature genes at E16.5. (A)** Heatmap, from Figure 1, showing the 72 downregulated genes after 9 hrs. FGFR2b ligand inhibition. These genes comprise the FGFR2b signature at this time-point. **(B)** Epithelial-specific *in situ* hybridization expression patterns from E14.5 embryonic sections retrieved from the GenePaint database (https://gp3.mpg.de/). Only a small number of the 72 regulated genes were found in the database, and of those only 13 showed clear epithelial expression patterns.

**
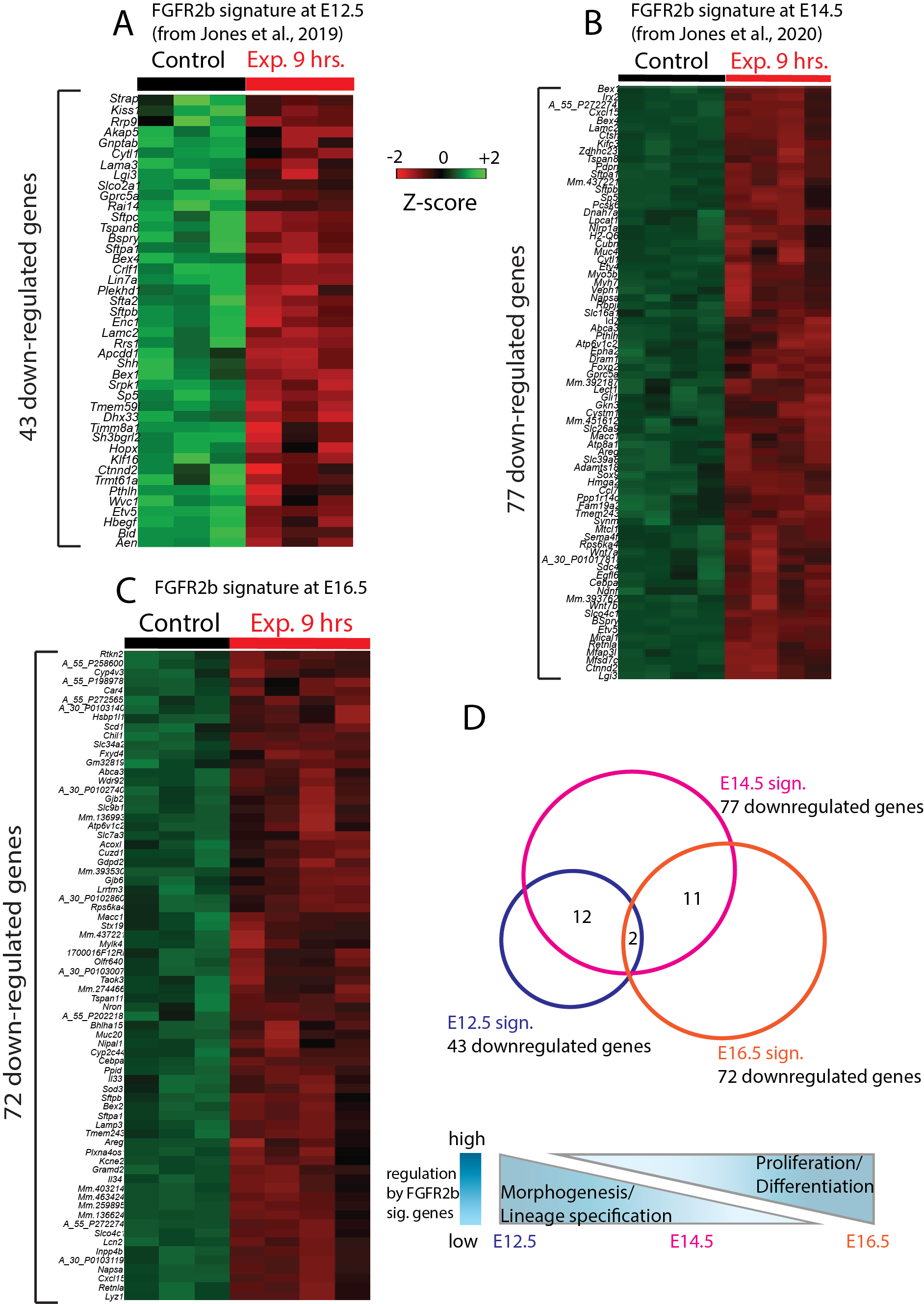
**

**Figure S6: Transcriptional targets of FGFR2b signalling during early and mid-pseudoglandular development. (A)** Heatmap of the 43 downregulated genes at E12.5 after 9 hours FGFR2b ligands inhibition, constituting the E12.5 FGFR2b signature (adapted from Jones et al. [13]). **(B)** Heatmap of the 77 downregulated genes at E14.5 after 9 hours FGFR2b ligands inhibition, constituting the E14.5 FGFR2b signature (adapted from Jones et al. [14]). **(C)** Heatmap of the 72 downregulated genes at E16.5 after 9 hours FGFR2b ligands inhibition, constituting the E16.5 FGFR2b signature. **(D)** Venn diagram showing the shared genes among the three gene signatures. The E12.5 signature shares 12 genes with the E14.5 signature. The E14.5 signature shares 11 genes with the E16.5 signature. Two genes (*Sftpa1* and *Sftpb*) are shared among the three signatures. These three signatures regulate shifting and overlapping biological activities over pseudoglandular development: branching morphogenesis and lineage specification which gives way to proliferation and differentiation later on.

**
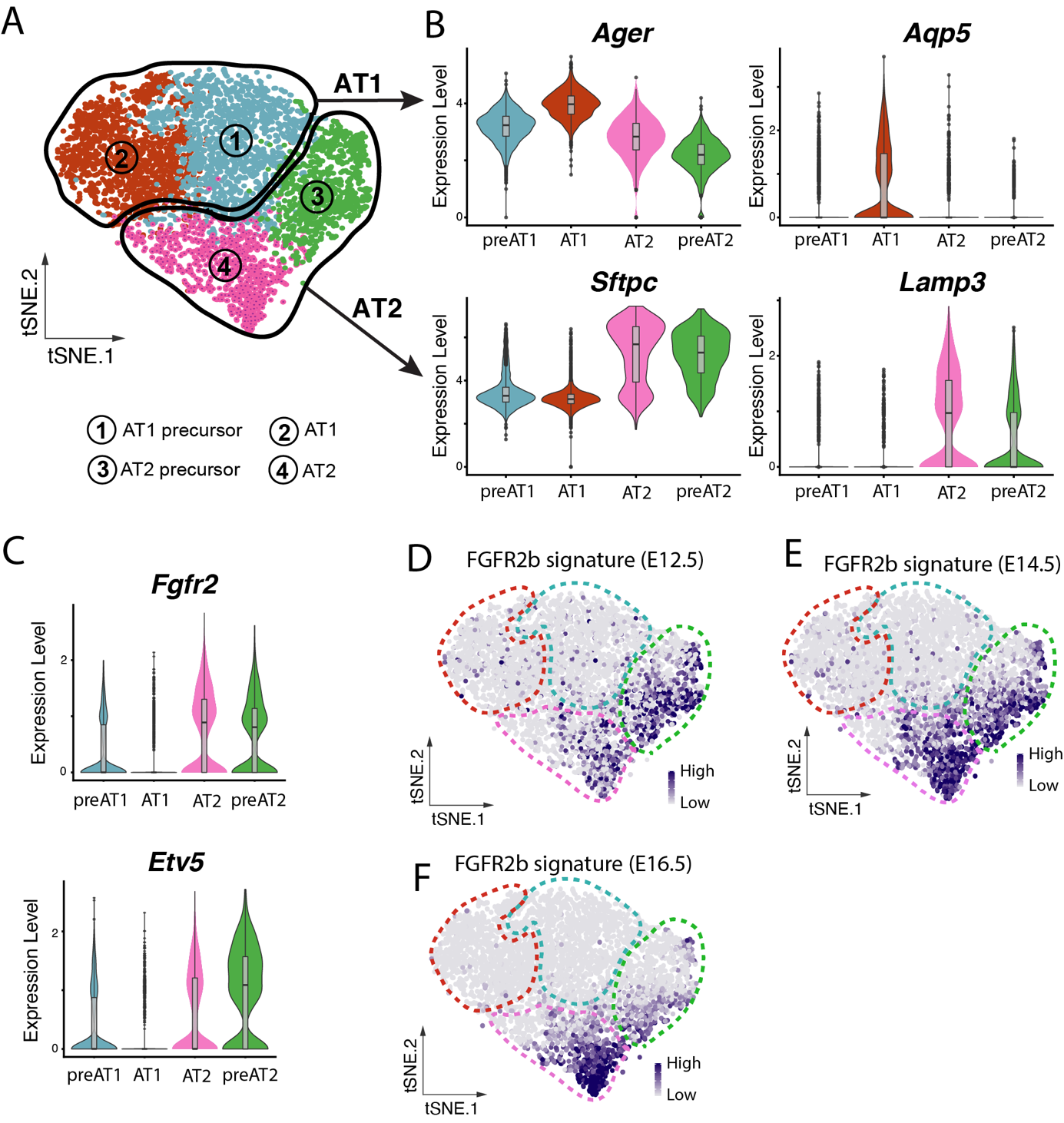
**

**Figure S7: Data-mining of scRNA-seq data from isolated E17.5 Nkx2-1-positive cells shows a narrowing of embryonic FGFR2b signatures to a subcluster of AT2 cells. (A)** Four clusters reproduced from Frank et al. [7] , GEO GSE113320: 1, blue – AT1 precursor; 2, red – mature AT1 cells; 3, green – AT2 precursor; 4, pink – mature AT2 cells. **(B)** Violin plots depict the expressions of AT2 (*Sftpc* and *Lamp3*) and AT1 markers (*Ager* and *Aqp5*) in the four clusters. **(C)** Violin plots of *Fgfr2* and the canonical downstream effector of FGFR2b signalling, *Etv5*, show that the two AT2 clusters, as well as to a lesser extent the AT1 precursors, show expression of these genes. **(D-G)** Reorientation of the four clusters from ‘A’ for ease of analysis. Expressions of FGFR2b signature genes at E12.5 (E), E14.5 (F), and E16.5 (G) reveal a narrowing of FGFR2b responsive cells to a subcluster of mature AT2 cells (cluster 4) and AT2 precursors (cluster 3).

**
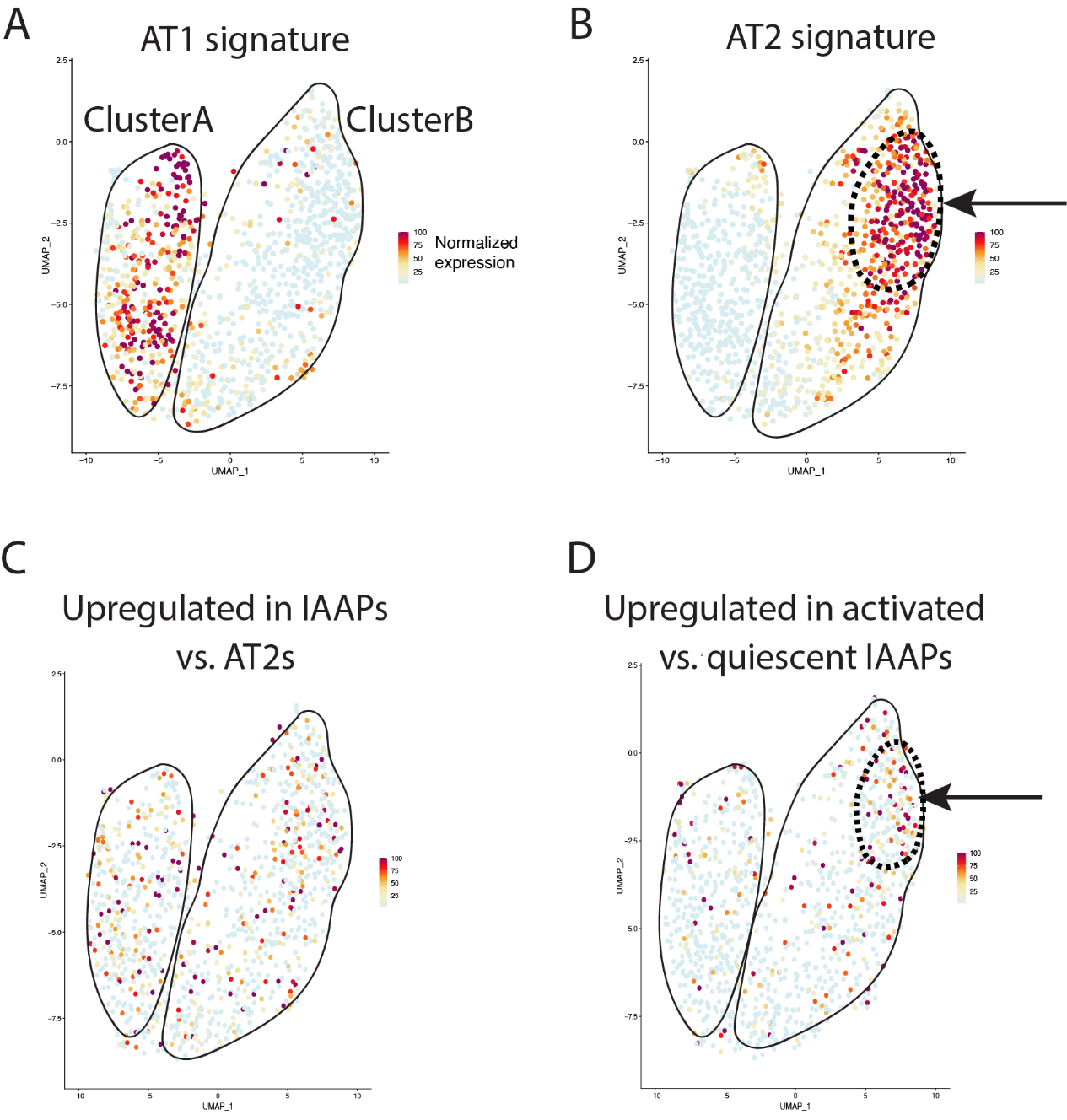
**

**Figure S8: Gene signatures delimit subclusters within mature AT2s.** Subclustering of the mature AT2 cluster from Frank et al. [7] (recall Fig. 6) reveals two subclusters, A and B. **(A)** AT1 signature genes are expressed almost exclusively by cluster A cells. **(B)** AT2 signature genes concentrate in a portion of cluster B cells (arrow and hashed line). **(C)** Genes upregulated in IAAPs compared to mature AT2 cells as provided by Ahmadvand et al. [19] (2021) are scattered throughout both clusters in our analysis. **(D)** Genes upregulated in activated IAAPs vs. quiescent IAAPs also scatter in both clusters, however, there seems to be a concentration of expression in a portion of cluster B, which overlaps with the cells expressing the AT2 signature (black arrows and hashed lines).


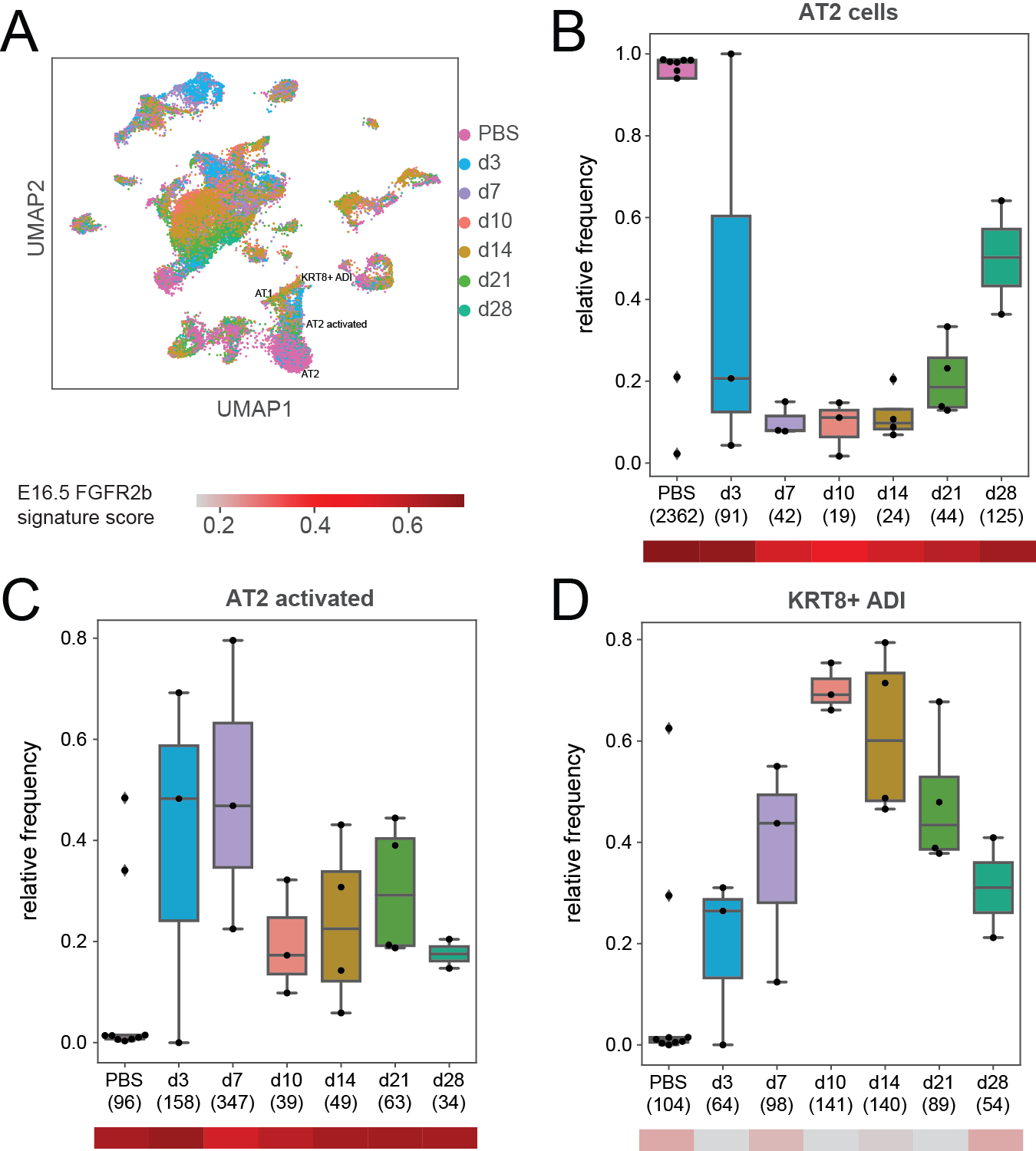


**Figure S9: The E16.5 FGFR2b signature is nearly lost in KRT8+ ADI cells during repair after bleomycin-induced lung injury. (A)** UMAP showing the single-cell data set from Strunz et al. 2020, with the alveolar lineage labeled (AT2, AT2 activated, KRT8+ ADI, and AT1 populations). **(B-D)** Box and whisker plots showing the frequency of each cell type (B: AT2 cells; C: AT2 activated cells; D: KRT8+ ADI cells) relative to the entire alveolar cell population over time. Numbers in brackets represent absolute cell numbers. Heatmaps show the expression scores of the E16.5 FGFR2b signature for each cell type at each time point.

**Table S1**: Primers for genotyping transgenic (Tg) and wildtype (WT) genomic sequences

| **Gene** | **Tg - Forward** | **Tg - Reverse** | **WT - Forward** | **WT - Reverse** | **Product size (bp)** |
| --- | --- | --- | --- | --- | --- |
| *Rosa26rtTA flox* | GAGTTCTCTGCTGCCTCCTG | CGAGGCGGATACAAGCAATA | AAGACCGCGAAGAGTTTGTC |  | Tg – 215  WT – 322 |
| *Tet(o)sFgfr2b* | CAGGCCAACCAGTCTGCCTGGC | CGTCTGAGCTGTGTGCACCTCC |  |  | Tg – 310 |
| *Sftpc-CreERT2* | CCCAGTCCCTCTCTGAATTTG | CATCGCTCGACCAGTTTAGTTA | GTTTCTACCGACCCTGTGAAG |  | Tg – 1000  WT – 500 |
| *Hopx-CreERT2* | CGAGGGGATCAGATGAAGAA | CCAAAAGACGGCAATATGGT | GCAGGACAGCAAAACAATGA |  | Tg – 800  WT – 396 |
| *Fgfr2bflox* | CTGCCTGGCTCACTGTCC | CTCAACAGGCATGCAAATGCAAGGTC |  |  | Tg – 480  WT – 380 |
| *tdTomatoflox* | CTGTTCCTGTACGGCATGG | GGCATTAAAGAGCGTATCC | CCGAAAATCTGTGGGAAGTC | AAGGGAGCTGCAGTGGAGTA | Tg – 196  WT – 297 |
| *Tet(o)caFgfr1* | GGCGTGTACGGTGGGAGGCCTATATAAGC | GAACGCCTCTGTGGAGACACGCGCGGCTCC |  |  | Tg – 325 |

**Table S2**: qPCR primers

| **Gene** | **Forward** | **Reverse** | **Product size (bp)** |
| --- | --- | --- | --- |
| *Fgfr2b* | CCTACCTCAAGGTCCTGAAGC | CATCCATCTCCGTCACATTG | 84 |
| *Sftpa1* | CAGTGTGATTGGGAGAAACCA | ATGCCAGCAACAACAGTCAA | 88 |
| *Sftpb* | GGCTAGACAGGCAAAAGTGTG | GACCGCGTTCTCAGAGGTG | 171 |
| *Hprt* | TCCTCCTCAGACCGCTTTTT | ATCATCGCTAATCACGACGC | 82 |
